# Unveiling the substrate specificity of the ABC transporter Tba and its role in glycopeptide biosynthesis

**DOI:** 10.1016/j.isci.2025.112135

**Published:** 2025-03-03

**Authors:** Nicola Gericke, Dardan Beqaj, Thales Kronenberger, Andreas Kulik, Athina Gavriilidou, Mirita Franz-Wachtel, Ulrich Schoppmeier, Theresa Harbig, Johanna Rapp, Iwan Grin, Nadine Ziemert, Hannes Link, Kay Nieselt, Boris Macek, Wolfgang Wohlleben, Evi Stegmann, Samuel Wagner

**Affiliations:** 1 Cellular and Molecular Microbiology, Interfaculty Institute of Microbiology and Infection Medicine (IMIT), University of Tübingen, Elfriede-Aulhorn-Str. 6, 72076 Tübingen, Germany; 2 Microbial Active Compounds, Interfaculty Institute of Microbiology and Infection Medicine (IMIT), University of Tübingen, Auf der Morgenstelle 28, 72076 Tübingen, Germany; 3 Partner-Site: DZIF Tübingen, Elfriede-Aulhorn-Str. 6/Auf der Morgenstelle 28, 72076 Tübingen, Germany; 4 School of Pharmacy, Faculty of Health Sciences, University of Eastern Finland, Yliopistonrinne 3, 70211 Kuopio, Finland; 5 Translational Genome Mining for Natural Products, Interfaculty Institute of Microbiology and Infection Medicine Tübingen (IMIT), University of Tübingen, Auf der Morgenstelle 24, 72076 Tübingen, Germany; 6 Proteome Center Tübingen, Institute of Cell Biology, University of Tübingen, Auf der Morgenstelle 15, 72076 Tübingen, Germany; 7 Excellence Cluster "Controlling Microbes to Fight Infections" (CMFI), University of Tübingen, 72076 Tübingen, Germany; 8 Interfaculty Institute for Bioinformatics and Medical Informatics (IBMI), University of Tübingen, Sand 14, 72076 Tübingen, Germany; 9 Bacterial Metabolomics, Interfaculty Institute of Microbiology and Infection Medicine (IMIT), University of Tübingen, Auf der Morgenstelle 28, 72076 Tübingen, Germany; 10 Microbiology/Biotechnology, Interfaculty Institute of Microbiology and Infection Medicine (IMIT), University of Tübingen, Auf der Morgenstelle 28, 72076 Tübingen, Germany

**Keywords:** Biosynthesis, Biochemistry, Chemical synthesis

## Abstract

Glycopeptide antibiotics (GPA) such as vancomycin are essential last-resort antibiotics produced by actinomycetes. Their biosynthesis is encoded within biosynthetic gene clusters, also harboring genes for regulation, and transport. Diverse types of GPAs have been characterized that differ in peptide backbone composition and modification patterns. However, little is known about the ATP-binding cassette (ABC) transporters facilitating GPA export. Employing a multifaceted approach, we investigated the substrate specificity of GPA ABC-transporters toward the type-I GPA balhimycin. Phylogenetic analysis suggested and *trans*-complementation experiments confirmed that balhimycin is exported only by the related type I GPA transporters Tba and Tva (transporter of vancomycin). Molecular dynamics simulations and mutagenesis experiments showed that Tba exhibits specificity toward the peptide backbone rather than the modifications. Unexpectedly, deletion or functional inactivation of Tba halted balhimycin biosynthesis. Combined with proximity biotinylation experiments, this suggested that the interaction of the active transporter with the biosynthetic machinery is required for biosynthesis.

## Highlights

• Tba recognizes balhimycin’s backbone, rather than its attached modifications
• The biosynthesis of balhimycin is dependent on a functional ABC transporter
• Proximity labeling suggests Tba interacts with the biosynthetic machinery

## Graphical abstract

**Figure:**
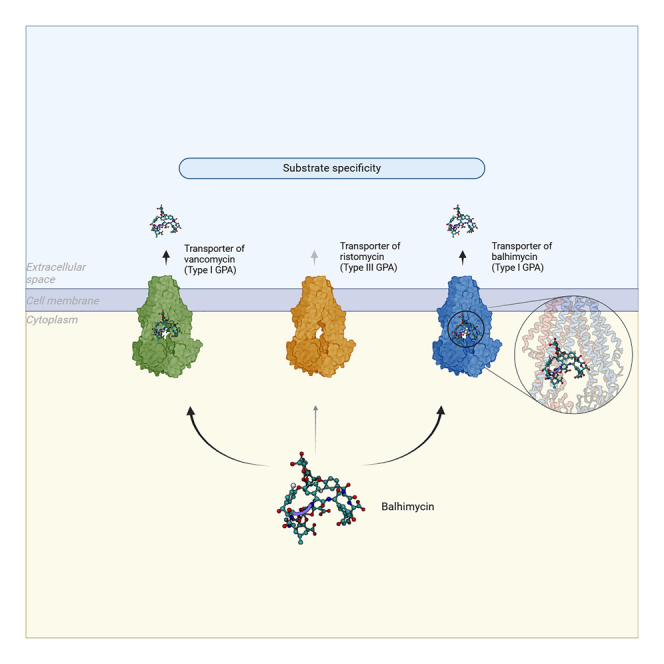

## Introduction

Glycopeptide antibiotics (GPAs) belong to a diverse class of bioactive compounds synthesized by various actinomycetes. Notable examples include vancomycin and teicoplanin, which are clinically used to treat infections caused by multidrug-resistant gram-positive bacterial pathogens.^1^

Based on their backbone composition, GPAs have been classified into five structural subclasses. Type I-IV GPAs are commonly referred to as “true” GPAs.^2^ They share a similar backbone composition, featuring type-specific amino acids (aa) at positions aa1 and aa3 with either aliphatic (I) or aromatic (II, III, IV) side chains. However, the modification pattern of the backbone varies between GPAs within the same type.^3^ Type V GPAs have recently been classified as glycopeptide-related peptides (GRPs) due to significant differences in structural, phylogenetic, and functional features.^4^ GPA backbones are synthesized by non-ribosomal peptide synthetases (NRPS)^5^ from both proteinogenic and non-proteinogenic aa. During peptide assembly the aromatic side chains are halogenated and cross-linked by dedicated enzymes, resulting in the antibiotic’s characteristic multicyclic and cup-shaped structure.^6^^,^^7^^,^^8^ Following peptide assembly, additional diversifications such as glycosylation, acylation, methylation, and sulfonation occur.^9^ The enzymes responsible for these modifications are typically encoded within biosynthetic gene clusters (BGCs).

The export of GPAs represents the last step in their biosynthesis process. To date, only one transporter associated with GPAs, the transporter of the type I (vancomycin type) GPA balhimycin (Tba) in *Amycolatopsis balhimycina* has been functionally characterized.^10^ This ATP-binding cassette (ABC) transporter specifically mediates the export of balhimycin and is not involved in self-resistance.^10^^,^^11^ ABC transporters form a large superfamily of integral membrane proteins and are known to facilitate ATP-driven transport of various substrates across the cytoplasmic membrane. However, they may fulfill other roles in addition to transport.^12^ ABC transporters can be associated with accessory domains,^13^ play a vital role in the biosynthesis of their substrates,^14^^,^^15^ or be involved in producer’s self-immunity.^16^^,^^17^ The latter is particularly relevant to ABC transporters within BGCs encoding compounds with antimicrobial activity.

Considering the paucity of data on how the processes of biosynthesis and export of GPAs are coordinated, we performed a comprehensive investigation of the specificity and functional roles of GPA-associated transporters, with a particular emphasis on the ABC transporter of balhimycin, Tba. Phylogenetic analysis, molecular dynamics simulation (MD simulation), and transport assays revealed that the specificity of Tba toward GPAs of the same type as balhimycin is dictated by the peptide backbone rather than the sugar modifications. Unexpectedly, deletion or functional inactivation of Tba did not lead to intracellular accumulation of balhimycin, suggesting a regulatory role of the transporter in balhimycin biosynthesis. This notion was supported by the observation that the transporter is in close proximity to the biosynthetic machinery.

## Results

### Bioinformatic analysis and phylogenetic reconstitution of GPA ABC transporters suggest type-associated specificity

Members of the ABC transporter family share a similar architecture consisting of two cytoplasmic nucleotide binding domains (NBD) responsible for ATP binding and hydrolysis, coupled with two transmembrane domains (TMD) forming the substrate translocation pathway at their mirror axis.^18^ Based on these features, we searched for putative ABC transporter genes in 89 GPA and GRP BGCs from producers of various families such as *Pseudonocardiaceae*, *Streptosporangiaceae*, *Micromonosporaceae*, *Streptomycetaceae*, and *Nocardiaceae.* Our analysis identified a single gene encoding a putative transporter in each BGC (Table S1).

Analysis of transmembrane topology based on prediction of the membrane integration propensity of segments of the protein sequence,^19^ revealed six hydrophobic regions in the N-terminal half of all scanned proteins (exemplary in Figure S1). Furthermore, typical motifs for functional NBDs -Walker A, Walker B, and ABC signature motif (C-loop) as well as A-, Q-, D loop, and H-switch^18^- were identified in the C-terminal half of every protein. These findings strongly suggest that the GPA BGCs encode a homodimeric ABC transporter with six transmembrane (TM) helices per TMD, wherein each half-transporter consists of one TMD and one NBD, similar to Tba.^10^ All transporters shared an aa sequence identity of more than 50% (Figure S2). Notably, sequence identities between transporters from putative type I-IV GPA BGCs were substantially higher than those to the transporters of GRP BGCs (Figure S2). Transporters with the highest similarity to Tba (88% sequence identity) were identified in *Amycolatopsis orientalis* DSM 40400, *Amycolatopsis keratiniphila* HCCB10007, and *Amycolatopsis regifaucium* DSM45072. These strains harbor BGCs for vancomycin (the first two)^20^^,^^21^ and decaplanin,^22^ both of which, like balhimycin, are type I GPAs.

**Figure 1:**
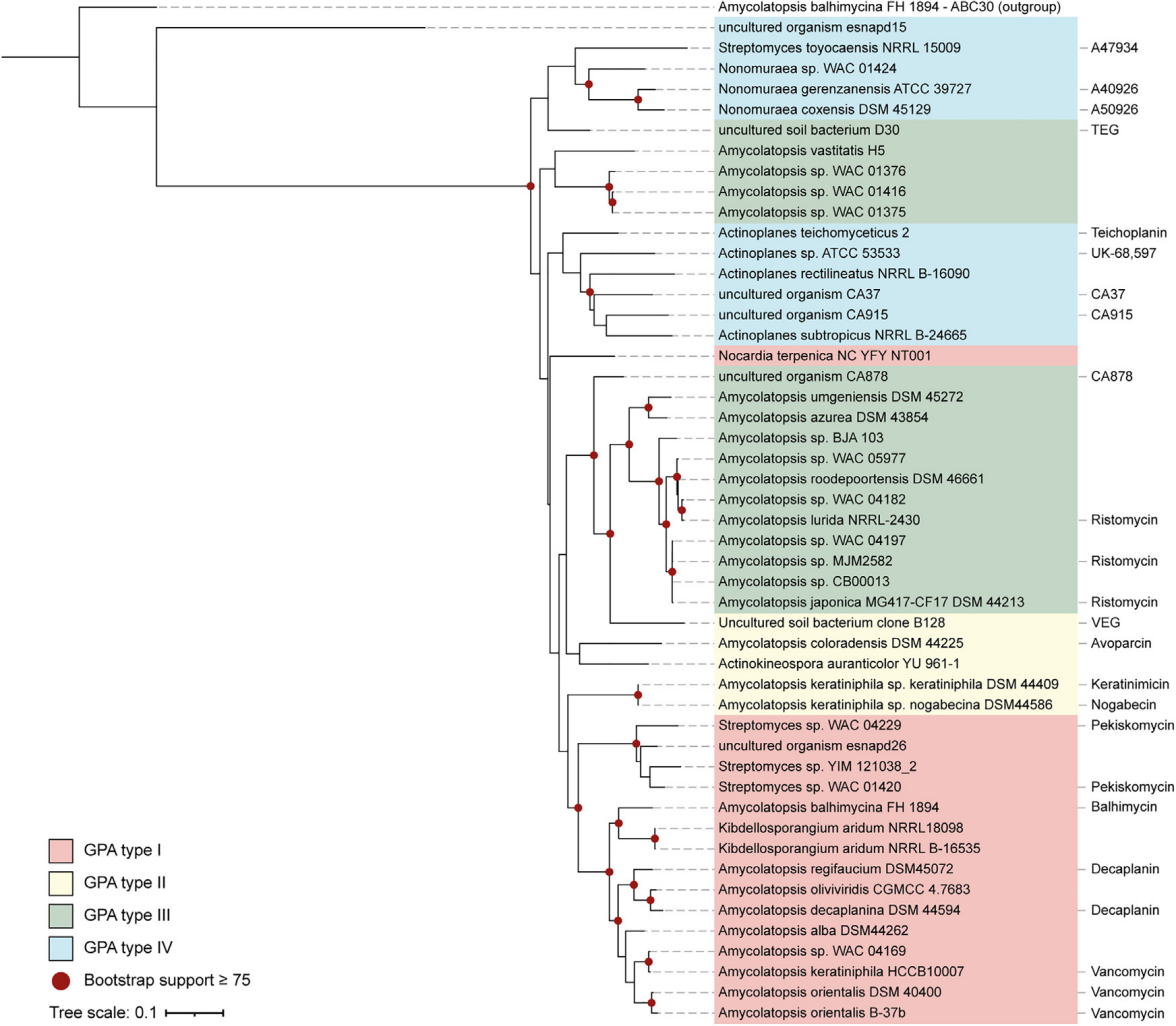
Evolutionary relationship within the group of GPA ABC transporters Phylogenetic tree (maximum likelihood (LG + F + G) algorithm with 1,000 bootstrap repetitions) of ABC transporters encoded in BGCs of GPAs. Nodes with bootstrap support ≥75% are marked by red dots on the branches. The tree was rooted using a putative ABC transporter encoded in an uncharacterized BGC in A. balhimycina (Abc30). The strain from which the BGC and thus the associated ABC transporter originates is indicated in the tree. (Predicted) GPA types are labeled accordingly: type I (red), type II (yellow), type III (green), type IV (blue). Known and predicted GPAs are mentioned behind the branches.

To determine the substrate spectrum of the identified transporters, we investigated their evolutionary relationship with GPAs. Using all 89 transporter sequences, we generated a phylogenetic tree employing the maximum likelihood algorithm (Figure S3), with an unrelated ABC transporter (Abc30) from *A. balhimycina* serving as an outgroup. By correlating the transporters with the GPA known GPA/GRP types I-V^4^ potentially encoded by the BGCs that also encode the respective transporter, we have categorized them according to these, creating a classification based on their substrates (in colors, Figure S3). Type V (GRP) transporters form a distinct cluster separate from all type I-IV transporters in the phylogenetic tree, indicating an earlier evolutionary divergence and significant differences in their putative substrates. Consequently, our focus was exclusively on type I-IV GPA transporters. Consistent with the high sequence identity, phylogenetic analysis of only type I-IV GPAs did not reveal distinct and widely separated clades, but a rough grouping of transporters associated with the export of GPAs of the same type, indicating an underlying specificity for them (in colors, Figure 1). Transporters exporting GPAs of the same type but with different modification patterns tended to cluster closely together (Figure 1). For example, balhimycin, decaplanin, and vancomycin, which share a type I backbone but vary in glycosylation patterns, formed a distinct clade (Bootstrap >81.6%). These findings indicate that the evolutionary trajectory of the transporters follows that of the BGCs, as these also build clades based on GPA type.^23^ They also suggest that the specificity of the transporters may be associated with the peptide backbone rather than its modifications.

### GPA-associated ABC transporters demonstrate specificity in exporting their native substrates

**Figure 2:**
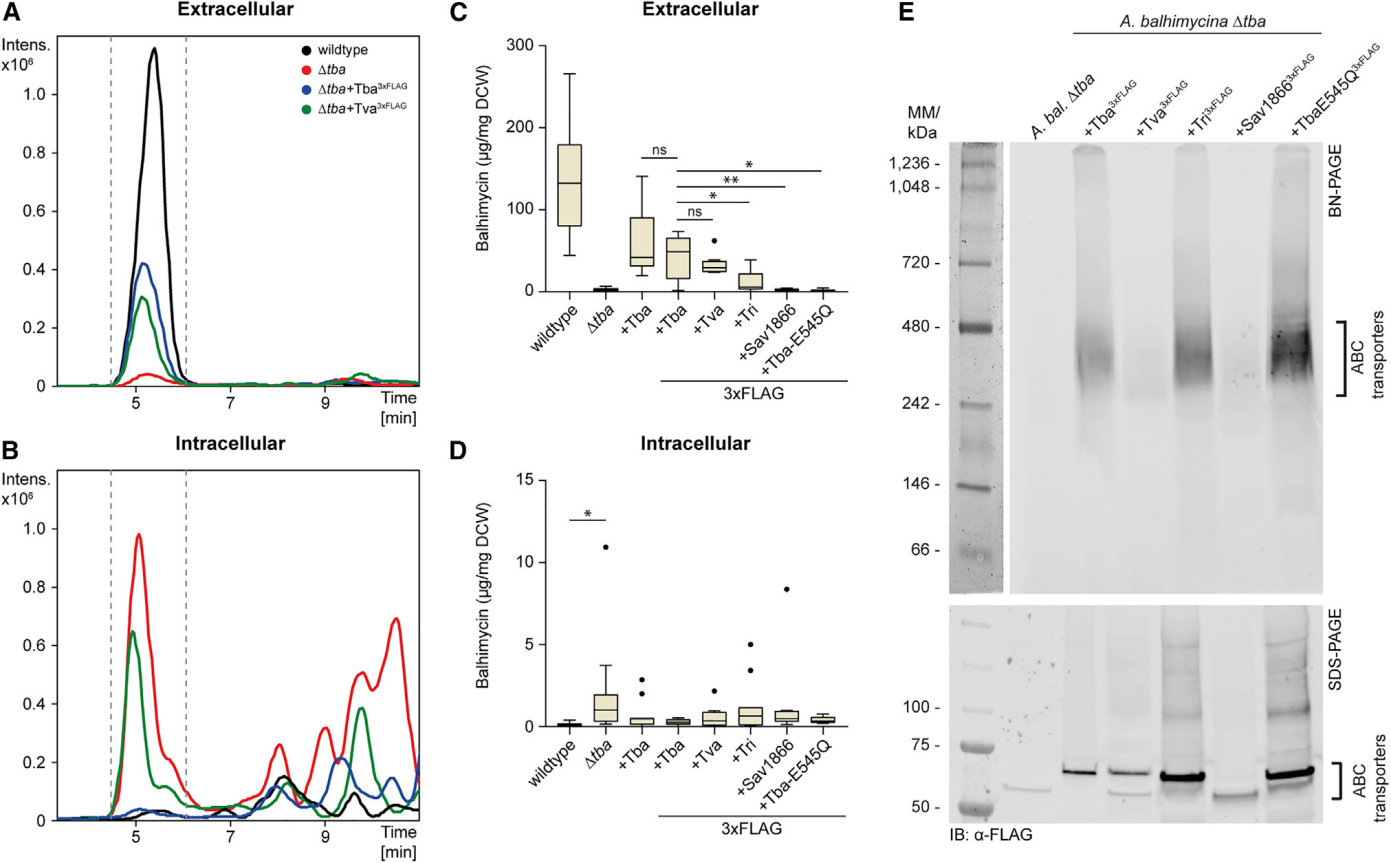
Analysis of complementation efficiency of *A. balhimycina* Δ*tba* using HPLC-MS detection of balhimycin (A and B) Raw (not normalized) HPLC-MS chromatograms of the culture supernatants (A) and mycelium extracts (B) of *A. balhimycina* wildtype (black), Δ*tba* (red), Δ*tba*+*tba*^*3xFLAG*^ (blue), and Δ*tba*+*tva*^*3xFLAG*^ (green). The peaks (retention time 5–5.5 min) represent the extracted ion chromatogram (EIC) of the protonated balhimycin mass *m/z* 1446.41 [M+H]^+^ (positive mode, smoothed 10.63–10.76 GA) (Figure S4). (C and D) Quantification of extracellular (C) and intracellular (D) levels of balhimycin in *A. balhimycina* and respective mutant strains based on HPLC-MS data. Values were normalized to the dry cell weight (DCW). All data are shown in boxplot representation. Error bars indicate standard deviations. Statistical outliers are displayed as dots. Statistically significant differences are highlighted with asterisks (*p* < 0.05 (∗); *p* < 0.01 (∗∗)) (“Wilcoxon signed-rank test” and “Benjamini-Hochberg procedure”) (Table S2). (E) Immunoblotting after BN- (top) and SDS-page (bottom) of solubilized crude membranes of *A. balhimycina* Δ*tba* and respective mutant strains. Detection with 3xFLAG specific antibodies.

*In silico* analyses and phylogenetic reconstitution of putative ABC transporters of GPAs suggested a significant degree of similarity among them. To empirically confirm their substrate specificity, we developed an *in vivo* system based on *trans* complementation of an *A. balhimycina* Δ*tba* mutant. To this end, we constructed the *A. balhimycina* Δ*tba* deletion and evaluated balhimycin export using high-performance liquid chromatography-mass spectrometry (HPLC-MS). Specifically, we quantified balhimycin in culture supernatants and mycelium extracts obtained from a 96-h culture of *A. balhimycina* wildtype and *A. balhimycina* Δ*tba* under balhimycin-producing conditions. The HPLC-MS results confirmed the presence of balhimycin in both strains, with prominent peaks corresponding to fully glycosylated (*m/z* 1587.49 [M+H]^+^), double-glycosylated (*m/z* 1446.41 [M+H]^+^), and mono-glycosylated (*m/z* 1305.34 [M+H]^+^) derivatives in each sample (Figures 2A, 2B and S4). Since accurate protein concentrations of the produced transporter could not be measured due to purification constraints, we relied on HPLC-MS results for quantification, normalizing to dry cellular weight for better comparability (Figures 2C and 2D).

The extracellular amount of balhimycin in *A. balhimycina* Δ*tba* was significantly reduced in comparison to *A. balhimycina* wildtype, by a factor of 50 (Figure 2C), in accordance with previous results.^10^ Unexpectedly, the level of balhimycin that accumulated intracellularly in *A. balhimycina* Δ*tba* was approximately 70-fold lower than the level of balhimycin exported by *A. balhimycina* wildtype, indicating impaired biosynthesis. To confirm that the reduction of balhimycin in *A. balhimycina* Δ*tba* resulted solely from the deletion of *tba*, we complemented the mutant with the native *tba* gene and with *tba*^*3xFLAG*^ under the control of a strong constitutive promoter (*ermE∗p*).^24^ Each gene was integrated into the genome at an ectopic locus, specifically the Φ*C31* attachment site. The expression of *tba* with a 3xFLAG epitope tag enabled us to verify the production and dimerization of the transporter via immunoblot analysis. Both complementations restored balhimycin export function in *A. balhimycina* Δ*tba* (Figure 2C), although not to wildtype levels, possibly due to the integration of the gene at a non-native locus. Statistical analysis revealed no significant difference between the amount of balhimycin exported by Tba or Tba^3xFLAG^ (Table S4), suggesting that the C-terminal epitope tag did not affect the export function of Tba.

**Figure 3:**
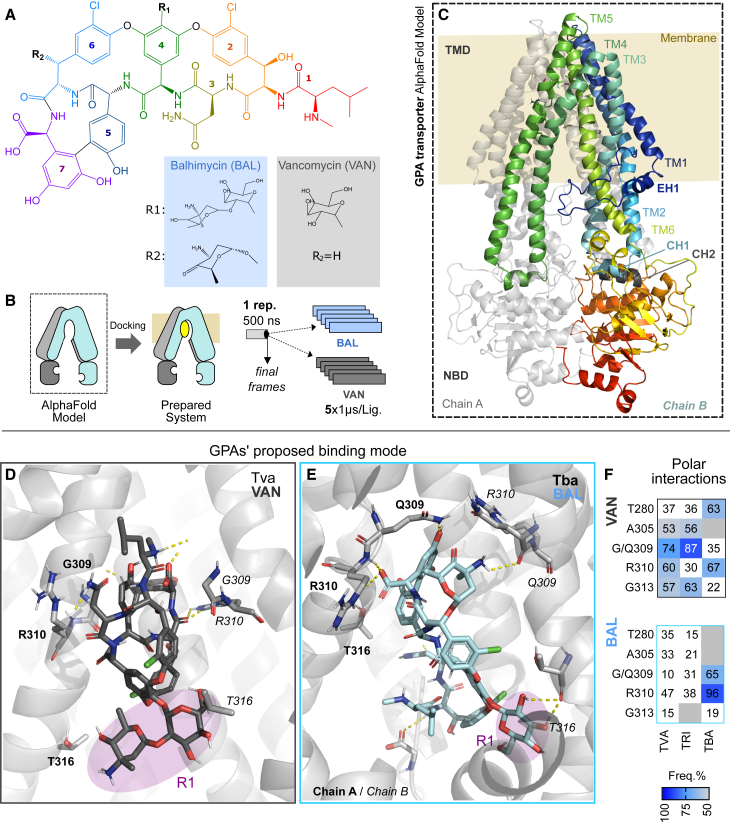
GPA binding mode on GPA transporter (A) 2D schematic representation of the GPAs exemplified by balhimycin and vancomycin, highlighting the individual portions and the insertion points for the sugar moieties. (B) Overview of the modeling pipeline (described in Star Methods 13). (C) 3D representation of a type IV GPA transporter, showing its six transmembrane (TM) helices (colored from dark blue to green) and the coupling (CH 1 and 2) and elbow (EH) helices. (D and E) Proposed binding mode for relevant GPAs, generated from representative conformations from the MD simulation for the transporter Tva bound to vancomycin (D) and Tba (E) bound to balhimycin. R1 substituent is highlighted in purple. Hydrogen bonds are depicted as yellow dashed lines. (F) heatmap representations of the hydrogen bond interaction frequencies in the analyzed trajectory. Numbering based on Tva sequence.

To investigate the substrate specificity of Tba, we complemented *A. balhimycina* Δ*tba* with genes coding for putative GPA transporters and a non-GPA transporter. We used transporters encoded in the BGC of vancomycin (Tva) from *A. keratiniphila* HCCB10007 and ristomycin (Tri) from *A. japonica* MG417-CF17 DSM 44213, both with a 3xFLAG epitope tag. Vancomycin (type I) differs from balhimycin only in the glycosylation pattern (Figure 3A), while ristomycin (type III) differs in the aa composition of the backbone and has a higher degree of glycosylation compared to balhimycin (Figure S5). All complemented mutants were compared with *A. balhimycina* Δ*tba*+*tba*^*3xFLAG*^ instead of the wildtype, ensuring a consistent starting point for analysis. Our results showed significant differences in the export of balhimycin by the ABC transporters used. Tva^3xFLAG^ exhibited a similar export to Tba^3xFLAG^, with no significant difference (*p* > 0.05). In contrast, Tri^3xFLAG^ exported significantly less (*p* = 0.03) balhimycin than Tba^3xFLAG^. Intracellularly, we observed only a slight accumulation of balhimycin in presence of Tva^3xFLAG^ or Tri^3xFLAG^ compared to Tba^3xFLAG^, but similar to *A. balhimycina* Δ*tba*, it was only detectable at very low levels (<1% of wildtype production). For complementation with a gene encoding a non-GPA ABC transporter, we selected the unrelated but well characterized multidrug transporter Sav1866 from *Staphylococcus aureus*,^25^ which shares only 33% sequence identity with Tba. In our assay, no export by Sav1866^3xFLAG^ was detected mirroring the findings from *A. balhimycina* Δ*tba*. Additionally, no significant intracellular accumulation was detectable.

The presence of the 3xFLAG epitope tag allowed us to verify proper production and insertion of the transporters into the membrane. This validation was crucial, particularly for transporters exhibiting minimal or no balhimycin export. All ABC transporters were detectable in the membrane fraction by immunodetection after SDS-PAGE (Figure 2E). Furthermore, BN-PAGE analysis following membrane solubilization with the nonionic detergent LMNG demonstrated that each transporter was detectable at the expected size (Figure 2E), indicating that neither aggregation nor incorrect dimerization significantly contributed to the reduced transport activity. However, the varying intensity of the bands indicated that the transporters differ in their accumulation levels in the membrane. For precise protein quantification, alternative methods should be considered in future studies to enable more accurate normalization and comparison of different transporters.

Altogether, the results of the *trans* complementation assay indicate that only the transporter gene from the BGC of a type I GPA, vancomycin, could complement *A. balhimycina* Δ*tba*, whereas the transporter gene from the BGC of a type III GPA, ristomycin could not. Given that vancomycin and balhimycin differ solely in their glycosylation pattern, we speculated that sugars may not play a pivotal role in binding to the ABC transporter, a hypothesis that we set out to validate.

### The specificity of the ABC transporter depends on the GPA backbone, rather than the sugar moieties

To further confirm the sugar independence and evaluate potential interaction between GPAs and ABCs, we applied a molecular modeling pipeline encompassing docking and long MD simulations (Figure 3B). We used AlphaFold2^26^ to generate structural models of all 89 ABC transporters. The predicted structures were nearly identical with differences only in the unstructured N-terminal region. The models contained all the previously described structural elements of type IV ABC transporters (Figures 3C and S12).^27^ Models featured a short N-terminal cytosolic helix (elbow helix, (Figures 3C and S12), a conserved TMD core comprising six TM helices, of which two perform a domain swap with those of the other half-transporter, and two coupling helices (i.e., CH1+2), responsible for the interaction of the TMD.^27^

We docked the type I GPAs vancomycin and balhimycin (Figure 3A) within a predicted binding cavity in the center of the inward-open TMD core (Figure 3D). These proposed binding modes underwent MD simulations and had their protein-ligand interaction frequencies monitored along the trajectory. Representative structures from the simulations displayed conserved interactions between the GPAs’ backbone and residues from the TMD core pocket, specifically, GPA aa at position 4–7 (Figures 3D and 3E) interacting with F269 (aa 6, numbering as in Figures 3A–3F, R310 (aa 5 and 7), G309, and G313 (aa 4). Interestingly, no significant differences were observed in the interaction pattern for aa 1–3, which are primarily responsible for the classification of the GPAs. We highlight this as a limitation of our current binding model. Additionally, in the proposed binding mode the R1 sugar moieties of GPAs point to the cytoplasm (Figures 3D and 3E). Based on that, we suggest that the sugar would contribute more to the compound solubility than to the binding energy.

### *In vivo* validation of binding interactions between tba and balhimycin confirms binding of the peptide backbone

Our MD simulation suggested that the transporter’s binding affinity toward the GPA substrates relies on interactions with the backbone. Notably, our model points to polar contacts between specific TMD residues and the GPA backbone, prompting us to validate the effect of mutations on transport efficiency.

**Figure 4:**
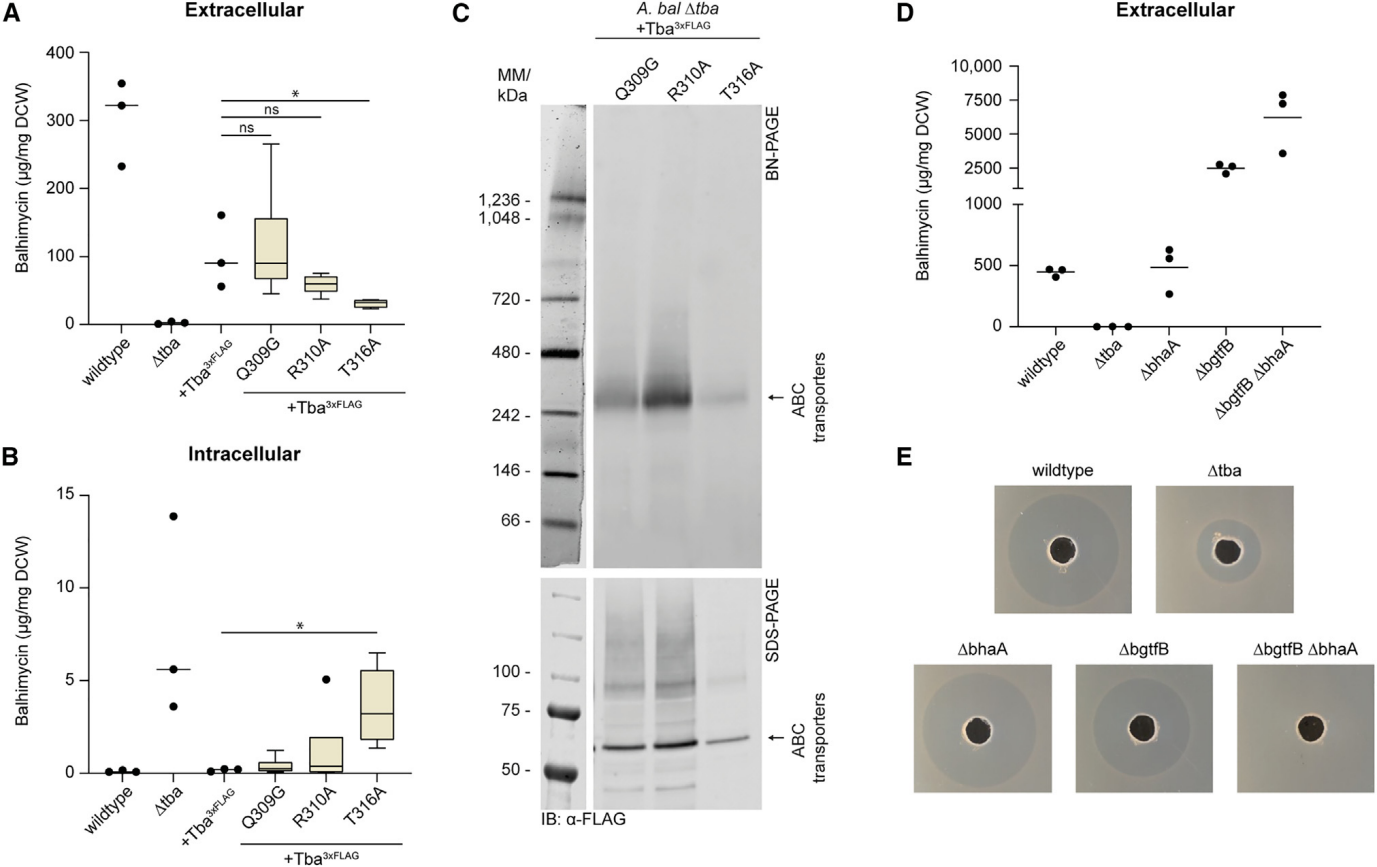
Analysis of transport specificity of the balhimycin transporter Tba (A and B) Quantification of extracellular (A) and intracellular (B) levels of balhimycin in *A. balhimycina* and respective mutant strains based on HPLC-MS data (Figure S6A). Values were normalized to the dry cell weight (DCW). Data are shown in boxplot representation or by individual data points (+median) if *n* ≤ 3. Error bars indicate standard deviations. Statistical outliers are displayed as dots as well. Statistically significant differences compared to +Tba^3xFLAG^ are highlighted with asterisks (*p* < 0.05 (∗)) (“Wilcoxon signed-rank test” and “Benjamini-Hochberg Procedure”) (Table S2). (C) Immunoblotting after BN- (up) and SDS-page (down) of solubilized crude membranes of *A. balhimycina* transporter mutant strains. Detection with 3xFLAG specific antibodies. (D) Quantification of extracellular levels of balhimycin in *A. balhimycina* and respective mutant strains based on HPLC-MS data (Figure S6B). Values were normalized to the DCW. Data are shown by individual data points (+median). (E) Bioassay on *B. subtilis* DSM10 indicator plates using culture supernatant of the indicated *A. balhimycina* strains.

To address this, we constructed mutants of *A. balhimycina* to probe the impact of Tba mutations on the transport process. Specifically, we introduced mutations in the TMD of the transporter Tba^3xFLAG^ at residues Q309 (G309 in Tva), R310 (conserved in all studied transporters), and T316 (conserved in GPA I-III transporters and located at the base of the substrate binding pocket), substituting them with either glycine or alanine (designated Q309G, R310A, and T316A, respectively). Complementation of *A. balhimycina* Δ*tba* with these constructs resulted in three recombinant strains carrying the individual mutations. Interestingly, only the T316A substitution significantly decreased the export level compared to the transporter Tba^3xFLAG^, emphasizing the critical role of T316 in the export process. Similar to a strain lacking Tba (Figures 2C and 2D), the *A. balhimycina* recombinant strain carrying the TbaT316A mutant slightly accumulated intracellular balhimycin, however, approximately 85-fold lower than the exported level by the wildtype. In contrast, the Q309G and R310A substitutions neither reduced the extracellular balhimycin levels nor had an effect on intracellular levels. (Figures 4A and 4B; Table S2; Figure S6A). Although T316 is not directly involved in balhimycin binding according to our model, its location on the cytoplasmic side of the putative substrate binding pocket suggests a role in pocket formation and stabilization. Importantly, we ruled out that the reduction in export levels was due to protein misfolding, as TbaT316A^3xFLAG^ was detected at the correct size by SDS-PAGE and BN-PAGE, albeit at lower accumulation levels than the wildtype protein (Figure 4C).

Our MD simulations suggest that the R1 sugar moieties do not interfere with binding (Figure S7), prompting us to assess the effects of GPA modifications on transport efficiency. In particular, we aimed to determine if non-glycosylated and non-chlorinated balhimycin could be exported by Tba. To investigate this, we used an *A. balhimycina* mutant lacking the halogenase gene *bhaA*, responsible for chlorination of the β-hydroxytyrosine residues at aa 2 and 6 of the balhimycin backbone^28^ (Figure 3A), and a mutant lacking the glycosyltransferase gene *bgtfB*, responsible for attaching the first sugar moiety to the hydroxyphenylglycine residue at AA4 (R1, Figure 3A). The deletion of *bgtfB* results in the production of a non-glycosylated heptapeptide, as glycosylation occurs stepwise in a defined order. Missing the first glycosylation step prevents the attachment of subsequent sugars.^29^ In addition, we included a double mutant lacking both genes, *bhaA* and *bgtfB*. By analyzing the supernatants via HPLC-MS we identified the peaks of the corresponding final products of each mutant and used the intensities for subsequent quantification. The HPLC-MS analysis of culture supernatants from *A. balhimycina* Δ*bhaA* showed peaks corresponding to masses of *m/z* 1237.5 [M+H]^+^ and *m/z* 1377.49 [M+H]^+^ (Figure S6B; Table S3), assignable to balhimycin without chlorine and with either one or two sugar moieties, respectively. In the culture supernatant of *A. balhimycina* Δ*bgtfB*, a peak with a corresponding mass of *m/z* 1142.28 [M+H]^+^ (Figure S6B; Table S3) was identified as chlorinated balhimycin lacking all sugar moieties. The double mutant exhibited peaks with prominent masses of *m/z* 1074.36 [M+H]^+^ and *m/z* 1088.38 [M+H]^+^, corresponding to balhimycin without chlorine atoms and sugar moieties, and the same molecule with an additional methyl group, respectively (Figure S6B; Table S3). All data were confirmed by comparing the UV-spectra derived from the diode array detector (DAD) with in-house databases (Figure S8).

Interestingly, the absence of sugar modifications on the balhimycin peptide backbone led to a significant increase in exported balhimycin derivatives, approximately 5.5-fold higher in *A. balhimycina* Δ*bgtfB* as compared to the glycosylated balhimycin in the wildtype (Figure 4D). However, the biological activity decreased in the absence of the sugars, as we confirmed by exposing the indicator strain *Bacillus subtilis* DSM10 to the culture supernatants of the *A. balhimycina* mutants (Figure 4E). Moreover, we observed an even higher export of balhimycin derivatives in the *A. balhimycina* double mutant Δ*bhaA*Δ*bgtfB*. The biological activity of the balhimycin derivative produced by this mutant was totally abolished (Figure 4E), which confirms the importance of these modifications in the mode of action of balhimycin. In *A. balhimycina* Δ*bhaA* the balhimycin derivative lacking chlorine atoms appeared to be exported at the same levels as fully modified balhimycin in the wildtype strain (Figure 4D), and didn’t exhibit any decrease in inhibitory activity. This indicates that chlorine atoms do not interfere with the transport process.

### The ABC transporter tba interacts with the GPA biosynthetic machinery at the membrane

**Figure 5:**
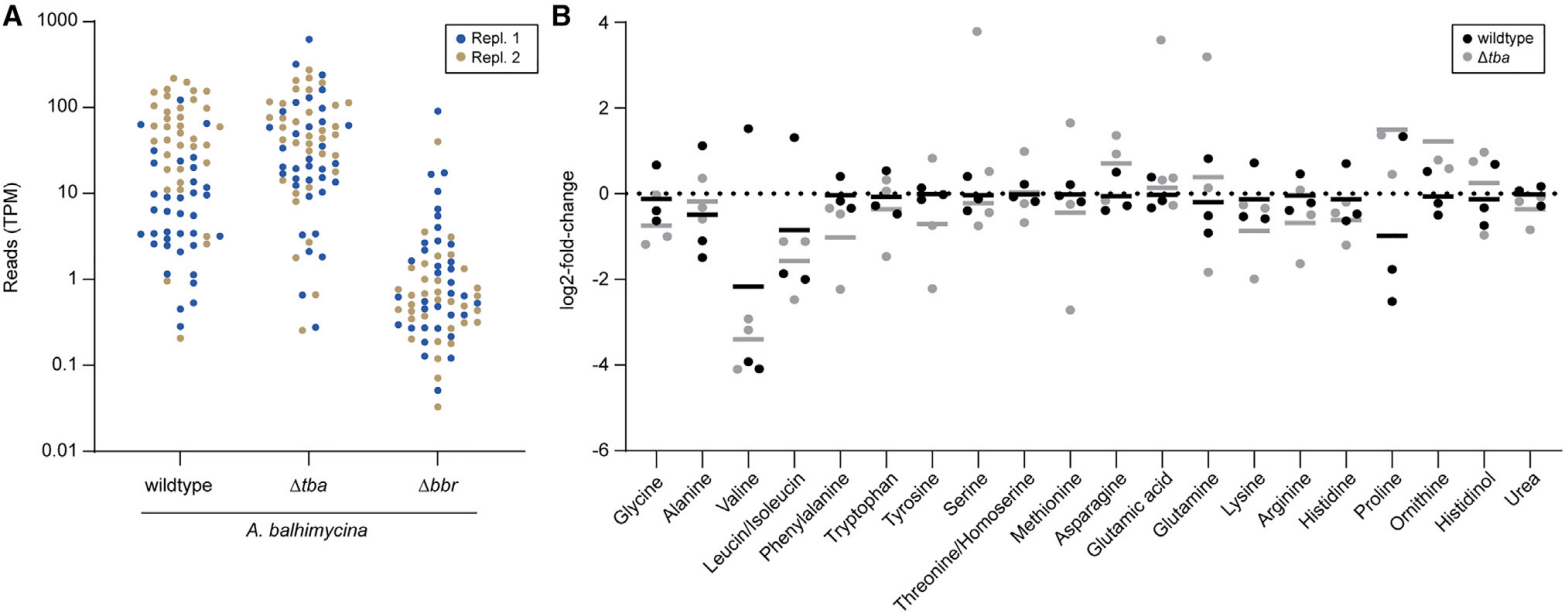
Transcriptome and metabolome analysis of *A. balhimycina* Δ*tba* (A) Display of transcription of every gene within the balhimycin BGC in *A. balhimycina* wildtype, Δ*tba*, and Δ*bbr* in transcripts per million (TPM). Two individual replicates are shown in beige and blue, respectively. (B) Amino acids levels after 48 h measured by LC-MS/MS and normalized to the dry cell weight (DCW). *A. balhimycina* wildtype (black) and Δ*tba* (gray) are displayed as log2 fold changes in comparison to a 13C internal standard.

The low intracellular balhimycin levels in the *A. balhimycina* Δ*tba* mutant and in the mutant strain producing the export-defective transporter TbaT316A^3xFLAG^ (Figures 2D and 4B) were unexpected. We anticipated intracellular accumulation of balhimycin following transporter deletion or inactivation, considering that *A. balhimycina* produces intrinsically resistant cell wall precursors. To confirm that active transport through Tba is required for the biosynthesis of balhimycin to occur, we constructed a mutant expressing TbaE545Q^3xFLAG^, a variant that carries a point mutation in the NBD, leading to a catalytically inactive protein unable to hydrolyze ATP. In this mutant, we could only detect negligible amounts of balhimycin both intracellularly and extracellularly (Figures 2C and 2D). This shows that TbaE545Q^3xFLAG^ is indeed unable to export balhimycin. Furthermore, the mere presence of the transporter is not sufficient to promote balhimycin biosynthesis, which would lead to its intracellular accumulation. Thus, it appears that a regulatory mechanism responsible for the arrest of balhimycin biosynthesis is triggered by the lack of active transport. When not actively exported, balhimycin could negatively regulate the transcription of the biosynthetic genes, thereby decreasing biosynthesis. We conducted a differential expression analysis of the transcriptome of *A. balhimycina* wildtype and *A. balhimycina* Δ*tba* to test this hypothesis (Figures 5A and S9A). We included in our investigation the *A. balhimycina* Δ*bbr* mutant lacking the StrR-like regulator gene *bbr*. Bbr is known to activate the transcription of the BGC genes.^30^ No significant difference in BGC expression was observed between the wildtype and *A. balhimycina* Δ*tba* after 48 h of cultivation. However, a clear downregulation was apparent in *A. balhimycina* Δ*bbr* (Figure 5A). This result shows that the absence of Tba does not affect the transcription of the genes in the BGC.

Since we observed no effect at the transcriptional level, we explored the possibility of biosynthesis inhibition due to the accumulation of precursors or intermediates of balhimycin biosynthesis acting as feedback inhibitors. By measuring the levels of aa and different balhimycin intermediates in *A. balhimycina* wildtype and Δ*tba* at the same time point (48 h) using LC-MS/MS and flow-injection mass spectrometry, respectively, we observed neither a significant increase or decrease in relevant aa (leucine, asparagine, and tyrosine) involved in balhimycin biosynthesis (Figure 5B), nor accumulation of intermediates (Figures S9B and S10).

**Figure 6:**
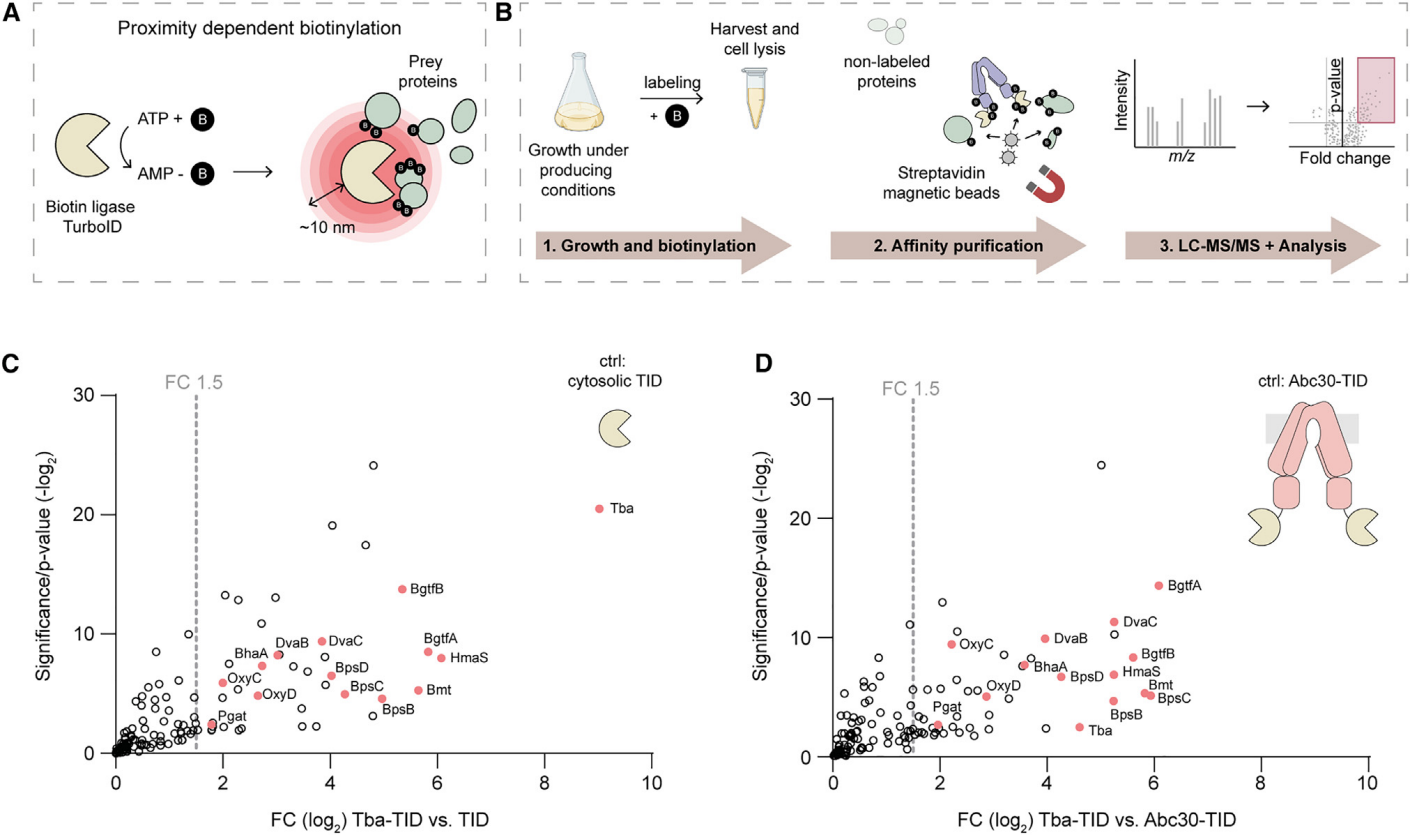
Identification of Tba’s microenvironment by proximity dependent biotinylation (A) Schematic overview of the concept of proximity dependent biotinylation by the promiscuous biotin ligase TurboID. B: Biotin. (B) Schematic representation of the experimental procedure of proximity dependent biotinylation in *A. balhimycina*. (C and D) Representation of all log2 fold change positive proteins detected by LC-MS/MS after proximity dependent biotinylation. Hits of Tba-TID compared to cytosolic TID (C) or membrane protein Abc30 (D). Proteins detected and encoded in the BGC of balhimycin are colored in salmon. NRPSs (BpsB and BpsC), modification enzymes (OxyC, BhaA, BgtfAB, and Bmt), and enzymes involved in precursor supply (Pgat, BpsD, OxyD, HmaS, DvaB, and DvaC).

Both transcriptome and metabolome analyses have remained inconclusive regarding the cause of the inhibition of balhimycin biosynthesis in the absence of the transporter. To explore the possibility that the transporter might interact with the biosynthetic machinery and thereby exert a direct regulatory function, we sought to identify the proteins localized in the vicinity of the transporter. To address this question, we employed a proximity dependent biotinylation approach using the recently developed and promiscuous biotin ligase TurboID (TID).^31^ Fusing TID to any protein of interest results in short-range (∼10 nm) biotinylation of primary amines on lysine residues of proximal proteins, thereby allowing the identification of the microenvironment of specific proteins^32^ (Figure 6A). We successfully established this method in *A. balhimycina DSM 44591* by using Tba as a bait protein for proximity studies (Figure 6B). We generated a recombinant strain by introducing a Tba^TID^ fusion construct into *A. balhimycina* Δ*tba* and confirmed that production of Tba^TID^ restored balhimycin export (Figure S11A). In order to detect biotinylation that is specific for Tba^TID^, we constructed two control strains of *A. balhimycina*. One strain produces TID as a cytosolic protein, while the other strain produces a GPA-unrelated transporter of *A. balhimycina*, Abc30, fused to TID. To enrich and identify the proteins biotinylated by Tba^TID^ we performed pull-downs with streptavidin beads (Figure S11B), followed by HPLC-MS/MS analysis. This yielded 563 proteins after processing the data. To ensure robust statistical analysis, we excluded hits identified in only one replicate. Intriguingly, comparison between the Tba^TID^ and control strains revealed a clear enrichment of balhimycin biosynthetic enzymes. These included NRPSs (BpsB, and BpsC), modification enzymes (OxyC, BhaA, BgtfAB, and Bmt), and enzymes involved in precursor supply (Pgat, BpsD, OxyD, HmaS, DvaB, and DvaC). The presence of these biosynthetic enzymes in close proximity to the Tba transporter suggests that an active transporter might “stimulate” the production of balhimycin via interaction with the biosynthetic machinery, a hypothesis that awaits confirmation in follow-up studies.

## Discussion

Our in-depth study of GPA-related ABC transporters provides valuable new insights into their evolutionary relationships, substrate specificity, and regulatory functions in GPA biosynthesis. The phylogeny of these transporters is consistent with the evolutionary trajectory of their respective substrates, as proposed by Hansen et al. and Waglechner et al. It is suggested that type I GPAs evolved from type IV GPAs.^23^^,^^33^ Consequently, our analysis implies that the putative ABC transporters of type IV GPAs are more ancestral than those of type I GPAs. Notably, transporters encoded by BGCs of the same GPA type often cluster together. This clustering suggests that their specificity is likely to be determined by differences in the aa composition of the GPA backbone rather than by variations in modifications, as GPAs of the same type can exhibit different modification patterns.

This hypothesis was further supported by MD-simulation and *in vivo* export analysis. Only the ABC transporters Tba and Tva, which are encoded in BGCs of type I GPAs, were found to export comparable quantities of balhimycin. In contrast, the putative ristomycin (type III GPA) ABC transporter Tri exported significantly less balhimycin. Our results suggest that the selectivity of GPA-associated ABC transporters is due to the chemical composition of the peptide backbone, since in ristomycin it consists exclusively of aromatic aa, whereas balhimycin has two aliphatic aa at position AA1 and AA3. We showed that Tba exports a non-glycosylated derivative of balhimycin at significantly higher levels than the fully glycosylated form. This suggests that the bulky and flexible sugar moieties, although crucial for biological activity, impedes efficient transport. There are examples of transporters in ribosomally synthesized and post-translationally modified peptides (RiPPs) produced by a wide range of bacteria that have been experimentally characterized. NisT, a transporter associated with the lantibiotic peptide nisin, is an example of a transporter with a broad substrate specificity.^34^ In contrast, the MjcD transporter exhibits high specificity for its cognate substrate, the lassopeptide Microcin J25.^35^ In contrast to RiPPs, the specificity of GPA-associated ABC transporters has been less well characterized. Our study reveals that GPA ABC transporters exhibit specificity toward their cognate substrates based on backbone composition, whereas they show greater promiscuity with respect to modifications of it.

In a previous study,^10^ we showed that *tba* deletion in *A. balhimycina* leads to accumulation of balhimycin, which stands in contrast to the findings of the current study. Here, we demonstrated that deletion or functional inactivation of the Tba transporter significantly reduces the amount of produced balhimycin. The discrepancy may be attributed to differences in the quantification method used; Menges et al. relied on inhibition zone diameters for quantification,^10^ whereas we employed state-of-the-art HPLC-MS analysis. Importantly, impaired biosynthesis in the *A. balhimycina tba* mutants is not due to toxic effects of balhimycin, as resistance is facilitated constitutively through cell wall precursor remodeling.^11^^,^^36^^,^^37^^,^^38^ Similar findings were reported previously, with deletion of transporter genes in particular BGCs leading to a reduction in the production levels of the respective compounds. Studies on the microcystin BGC in *Microcystis aeruginosa* showed that deletion of the transporter gene *mcyH* completely abolished the production of microcystin.^39^ Comparable observations were made in *Streptomyces ghanaensis* ATCC14672, in which the deletion of two transporter genes *moeX5moeP5* and *moeD5moeJ5* led to a significant reduction in the production of moenomycin A.^14^ Another example is provided by the NRPS derived compound cereulide and its cognate ABC transporter CesCD. The deletion or inactivation of CesCD resulted in a greatly reduced biosynthesis of cereulide by the producer strain *Bacillus cereus*.^15^ These studies point to an important role of the cognate transporters in regulating the biosynthetic machinery of natural products and strongly highlights active transport as a crucial factor for continuous biosynthesis.

To elucidate the mechanism behind transport-dependent balhimycin production, we investigated different levels of regulation and showed that transcription is not affected by the absence of Tba, as previously observed for microcystin biosynthesis in the absence of the cognate transporter.^39^ Feedback regulatory mechanisms have been reported in balhimycin biosynthesis, involving enzymes and intermediates from the shikimate-pathway.^40^^,^^41^ It was shown that the aromatic aa tyrosine and phenylalanine act as feedback enzymatic inhibitors of the shikimate pathway, thereby reducing the provision of balhimycin precursors. Our metabolomic analysis shows that none of the precursors accumulated in the *A. balhimycina* Δ*tba* deletion mutant, ruling out an inhibitory effect on biosynthetic enzymes. Furthermore, none of the intermediates of the NRPS assembly line appeared to be present at significantly higher concentrations. We conclude that feedback inhibition is not the reason for reduced biosynthesis of balhimycin in the absence of a functional transporter.

The results of our proximity dependent biotinylation experiment provide an alternative explanation: we showed that many of the enzymes responsible for assembly and modification of the balhimycin backbone as well as supply of precursors are in close proximity to Tba, possibly forming a microcompartment specialized in GPA production. Based on the general ABC transporter mechanisms,^18^ we speculate that upon substrate binding, Tba undergoes a conformational change that is required for stimulation of balhimycin biosynthesis. However, it remains elusive whether the transporter directly interacts with the biosynthetic enzymes and how exactly this interaction impacts balhimycin biosynthesis. There is evidence that biosynthetic enzymes of secondary metabolites interact with the corresponding transporter and that compound biosynthesis might be membrane-associated, as exemplified by the cereulide and nisin biosynthesis. Both biosynthetic machineries were shown to build a complex and co-localize with the corresponding ABC transporter.^15^^,^^42^^,^^43^

Among the biotinylated proteins with significant fold changes in the Tba^TID^ mutant, we identified a paraslipin protein, suggesting its close proximity to Tba and consequently to other proteins of the balhimycin biosynthetic machinery. Paraslipins belong to the stomatin, prohibitin, flotillin, and HflK/C (SPFH) superfamily and are known to be associated with the formation of functional membrane microdomains (FMM).^44^ Our finding suggests that Tba may be integrated into an FMM, serving as an anchor of biosynthetic enzymes. We speculate that the biosynthesis of balhimycin occurs within a specialized microcompartment and is likely regulated by the presence of catalytically active Tba, as suggested by our *in vivo trans* complementation assays. There is evidence that bacteria use microcompartments to optimize their metabolic processes. Carboxysomes are among the best studied and represent an example of how compartmentalization can increase the efficiency of metabolic reactions, in this specific case of carbon fixation by photoautotrophs and chemoautotrophs. Carboxysomes concentrate CO_2_ owing to the activity of a carbonic anhydrase and the selective permeability of the protein shell, which in turn increases both the rates and specificity of the encapsulated carbon-fixing enzyme ribulose 1,5-bisphosphate carboxylase/oxygenase (RuBisCO).^45^ Another example is the propanediol-utilizing microcompartment (Pdu MCP) of enteric bacteria like *Salmonella*. The Pdu MCP allows the degradation of 1,2-propanediol and enteric pathogenesis.^46^ However, further studies are required to confirm that in *A. balhimycina* GPA biosynthesis occurs in compartments associated with the membrane, and to fully elucidate the scope of the transporter’s potential roles in this process.

### Limitations of the study

Despite detailed investigation of the substrate specificity using *insilico* models, *in vitro* studies are essential to fully characterize the binding pocket of the ABC transporter Tba for the GPA balhimycin. Structural elucidation of Tba-balhimycin complex would provide crucial insights into the binding mechanism.

Additionally, the hypothesis that Tba interacts with the biosynthetic machinery requires further validation. Investigating the regulatory function of Tba through direct protein-protein interaction studies would be key to understanding its precise impact on the balhimycin biosynthesis.

## Resource availability

### Lead contact

Further information and requests for resources and reagents should be directed to and will be fulfilled by the lead contact, Evi Stegmann (evi.stegmann@uni-tuebingen.de)

### Materials availability

This study did not generate new unique reagents.

### Data and code availability

• All MD simulation trajectories, interaction data and MD quality control data, representative PDB files from the alphaFold models, phylogenetic trees and metabolomics raw data are available under the DOIs: https://doi.org/10.5281/zenodo.7547342, https://doi.org/10.5281/zenodo.7732071 and https://doi.org/10.5281/zenodo.7547403 and https://doi.org/10.5281/zenodo.14918266 are publicly available as of the date of publication.
• All RNA-seq Illumina read files as well as the raw counts have been deposited in NCBI’s Gene Expression Omnibus and are accessible under accession number GSE274067.
• The mass spectrometry proteomics data have been deposited to the ProteomeXchange Consortium via the PRIDE^47^ partner repository with the dataset identifier PXD054387.
• This study did not generate any unique code.
• Any additional information required to reanalyze the data reported in this paper is available from the lead contact upon request.

## Acknowledgments

The work in the laboratory of S.W., E.S., W.W., N.Z., B.M., and K.N. related to this study was funded by the 10.13039/501100001659Deutsche Forschungsgemeinschaft (DFG) as part of the transregional collaborative research center TRR 261 ‘Cellular Mechanisms of Antibiotic Action and Production’ (Projects B01/B02/B05/Z03). It was supported by the de.NBI Cloud within the 10.13039/501100018929German Network for Bioinformatics Infrastructure (de.NBI) and ELIXIR-DE (10.13039/501100003163Forschungszentrum Jülich and W-de.NBI-001, W-de.NBI-004, W-de.NBI-008, W-de.NBI-010, W-de.NBI-013, W-de.NBI-014, W-de.NBI-016, W-de.NBI-022). NGS sequencing methods were performed with the support of the 10.13039/501100001659DFG-funded NGS Competence Center Tübingen (INST 37/1049-1). Data management and storage of raw data for this project were supported by the Quantitative Biology Center (QBiC), 10.13039/501100002345University of Tübingen, Germany. The authors would like to thank Libera Lo Presti for her support in writing, comments on the manuscript, and fruitful discussions. S.W., E.S., W.W., T.K., and N.Z. acknowledges the 10.13039/100009139German Center for Infection Research (DZIF, TTU09.716). S.W., E.S., W.W., T.K., and N.Z. acknowledges funding by the Clusters of Excellence EXC2124 CMFI (project ID 390838134) and T.K. the TüCAD2 the Federal Ministry of Education and Research (BMBF) and the Baden-Württemberg Ministry of Science as part of the Excellence Strategy of the German Federal and State Governments – Germany, by the means of the program TüCAD_2_, as well as the 10.13039/100009139German Center for Infection Research (T.K.: DZIF, TTU06.716) (W.W. TTU09.826). The authors would like to thank the CSC—Finland for the generous provided resources, Ana Monica Daneliuc for contributing to refining HMM search for the creation of the BGC dataset and Anke Biedermann and Irina Droste-Borel from the Proteome Center Tübingen for technical support. Graphical abstract was created in BioRender. Stegmann, E. (2025) https://BioRender.com/x66x647. We acknowledge support from the Open Access Publication Fund of the University of Tübingen.

## Author contributions

N.G. performed *in silico* analysis of transporter sequences and phylogenetic analysis. N.G. and D.B. performed *in vivo* experiments and analyzed all data. T.K. performed MD simulations. S.W., E.S., and W.W. designed the study. N.G., D.B., T.K., S.W., E.S., and W.W. drafted the paper and wrote the original manuscript. A.K. performed HPLC-MS measurements. M.F.-W. and B.M. performed proteomics. A.G. identified BGCs and putative transporters and did the correlation of BGCs and substrates. U.S. did statistical analysis of *in vivo* data. J.R. and H.L. performed metabolomics. T.H. and K.N. performed RNA sequencing and analyzed the data. I.G. provided bioinformatic support.

## Declaration of interests

The authors declare no competing interests.

## STAR★Methods

### Key resources table

**Table:** 

REAGENT or RESOURCE	SOURCE	IDENTIFIER
**Antibodies**		

anti-FLAG® M2	Sigma-Aldrich	RRID:AB_259529
Rabbit polyclonal anti-BirA IgG	Thermo-Fisher	RRID:AB_2787583
goat anti-Mouse IgG DyLight™ 800	Thermo-Fisher	RRID:AB_2556774
goat anti-Rabbit IgG DyLight™ 800	Thermo-Fisher	RRID:AB_2556775
Streptavidin DyLight™ 800	Thermo-Fisher	RRID:AB_11152196

**Bacterial and virus strains**		

See Table S5 in the SI	This work	

**Chemicals, peptides, and recombinant proteins**		

Q5® Hot Start High-Fidelity DNA Polymerase	NEB	M0491L
Phusion® High-Fidelity DNA Polymerase	Thermo-Fisher	F-530XL
Taq DNA Polymerase	Thermo-Fisher	EP0401
*NdeI*	Thermo-Fisher	ER0585
*XbaI*	Thermo-Fisher	ER0683
*KpnI*	Thermo-Fisher	ER0522
*Apramycin*	Genaxxon	M3450.0005
*Ampicillin*	Roth	K029.4
*Erythromycin*	Roth	4166.2

**Deposited data**		

MD simulation trajectories, interaction data and MD quality control data, representative PDB files from the alphaFold models and phylogenetic trees	This work	https://doi.org/10.5281/zenodo.7547342 https://doi.org/10.5281/zenodo.7732071 https://doi.org/10.5281/zenodo.7547403
RNA-Seq Illumina read files as well as the raw counts	This work	GSE274067
Mass spectrometry proteomics data	This work	PXD054387
Metabolomics raw data	This work	https://doi.org/10.5281/zenodo.14918266

**Oligonucleotides**		

See Table S4 for cloning primers	This work	

**Recombinant DNA**		

See Table S6 for plasmids	This work	

**Software and algorithms**		

AlphaFold (v2.2.2)/Alphafold3	Jumper et al.^26^	
Desmond	Bowers et al.^48^	
OPLS4 force-field	Lu et al.^49^	
Glide	Friesner et al.^50^	
LigPrep	Shelley et al.^51^	
Maestro (v2022.4)	www.schrodinger.com	
prokka (v1.14.16)	Seemann^52^	
OrthoFinder platform (v2.3.11)	Emms and Kelly^53^	
blastP	https://blast.ncbi.nlm.nih.gov/	
scipy (v1.9.3)	Virtanen et al.^54^	
ΔG prediction server (v1.0)	Hessa et al.^19^	
MEGAX (v10.2.4)	Kumar et al.^55^	
MUSCLE algorithm	Edgar^56^	
iTOL (v6.6)	Letunic and Bork^57^	

**Other**		

Serva, NativePAGE™ 3 to 12% (Bis-Tris, 1.0 mm, Mini Protein Gels)	Thermo-Fisher	BN1001BOX
Immun-Blot® polyvinylidene difluoride (PVDF) membrane (0.2 μm)	Bio-Rad	#1620177

### Experimental model and study participant details

The bacterial strains and plasmids used in this study are described with the necessary information in Tables S5 and S6.

### Method details

#### Identification of transporter sequences of biosynthetic gene clusters

The biosynthetic gene clusters (BGC) of glycopeptide antibiotics (GPAs) were identified either by literature search^2^ or by a hidden Markov model (HMM) search in the NCBI database using the X-domain sequence of non-ribosomal peptide synthetases (NRPS) of known GPAs.^4^ If necessary, genomes and clusters were re-annotated with prokka (v1.14.16) using default parameters with specified genus.^52^ Subsequently, genes encoding putative ATP binding cassette (ABC) transporters were identified by using the OrthoFinder platform (v2.3.11).^53^ This tool classifies all genes into groups whose members are orthologous with each other and therefore suitable for phylogenetic analyses. The correlation of BGC to GPA types was done manually. Every gene in the BGC was blasted and studied in order to classify the BGC in one of the five classical types of GPAs (I-V). The criteria used were the predicted amino acids at positions one and three of the backbone, the number of P450 monooxygenase genes and the presence of an acyltransferase-encoding gene.

#### Bioinformatic analysis of transporters

Amino acid sequence identities of the transporters were calculated by blastP pairwise comparison and displayed in a heatmap representation. The order of the transporters is based on a hierarchical clustering calculated with the python library scipy (v1.9.3) and using the function "scipy.cluster.hierarchy.linkage". The algorithm used was “Weighted Pair Group Method with Arithmetic Mean” (WPGMA), which builds a hierarchy of the clusters (of similar transporters) using an agglomerative approach.^54^ The full protein scan option of the ΔG prediction server (v1.0)^19^ was used to identify putative transmembrane (TM) helices (full protein scan; helix length 19–23; +length correction). Common sequence motifs of the NBDs of the ABC transporters^18^ were manually identified after multiple sequence alignment (MSA) of all transporters using MEGAX (v10.2.4).^55^

#### Phylogenetic analysis of GPA associated ABC transporter

The evolutionary relationship between GPA associated ABC transporters was analyzed by phylogenetic tree construction. MSA, model testing, and calculation of the Maximum Likelihood phylogenetic tree were performed using the software MEGA-X (v10.2.4).^55^ Trimming of the MSA was done with the trimAl tool (v1.4.rev15 build[2013-12-17]) using default parameters,^58^ or manually. An unrelated ABC transporter (Abc30) of *A. balhimycina* was used as an outgroup. The phylogenetic tree was constructed as follows: all sequences were aligned using the MUSCLE algorithm^56^ and then used to analyze the best amino acid substitution model. The model testing identified as the most suitable the model of Le & Gascuel (LG)^59^ with frequencies (+F) and gamma distributed rates (number of discrete gamma categories = 5) (+G), and this was used by the ML algorithm with bootstrap test of 1000 repetitions to calculate phylogenetic trees. Visualization of phylogenetic trees was performed with iTOL (v6.6).^57^

#### Chemicals and reagents

Unless otherwise indicated, chemicals and enzymes were purchased from Sigma-Aldrich, Thermo-Fisher, and New England Biolabs. Primers listed in Table S4 were synthesized by Eurofins Scientific or IDT. Antibodies were purchased from Sigma-Aldrich (Mouse monoclonal anti-FLAG M2 (F3165)) and Thermo-Fisher (Rabbit polyclonal anti-BirA IgG (PA5-80251); goat anti-Mouse IgG DyLight 800 (SA5-35521); goat anti-Rabbit IgG DyLight 800 (SA5-35571); Streptavidin DyLight 800 (21851)). SERVAGel TG PRiME 8–16% precast gels were purchased from Serva, NativePAGE 3 to 12% (Bis-Tris, 1.0 mm, Mini Protein Gels) from Thermo-Fisher, and Immun-Blot polyvinylidene difluoride (PVDF) membrane (0.2 μm) from Bio-Rad.

#### Bacterial strains, plasmids, and growth conditions

All bacterial strains and plasmids used in this study are listed in Tables S5 and S6, respectively. *E. coli* was cultivated aerobically at 37°C in 10 mL tubes and shaken at 180 rpm in liquid LB medium (0.5% (w/v) yeast extract, 1% (w/v) tryptone, 85.56 mM NaCl) or on solid LB agar (1.5% (w/v)) medium. If necessary, the medium was supplemented with 100 μg/mL apramycin or 100 μg/mL ampicillin for plasmid selection. Unless otherwise indicated, *A. balhimycina* was cultivated aerobically at 29°C in baffled flasks with a steel coil, and shaken at 120 rpm in R5 medium^60^ or on the respective solid agar (1.5% (w/v)) medium. Fifty μg/ml apramycin or 50 μg/mL erythromycin was used for plasmid selection. All *A. balhimycina* strains in this study are derivatives of DSM 44591.

#### Molecular cloning

Different DNA modification techniques were used in this study. Polymerase chain reaction (PCR) with “Q5 Hot Start High-Fidelity DNA Polymerase” and “Phusion High-Fidelity DNA Polymerase” were used to amplify genes and DNA fragments, while “Taq DNA Polymerase” was used for colony PCR. pRM4^10^ vector DNA was linearized using the restriction endonucleases *NdeI/XbaI*, pSP1^61^ vector was linearized using the restriction endonuclease *KpnI*. Assembly of DNA fragments was performed using standard gibson assembly method.^62^ All primers used in this study are listed in Table S4.

#### Genetic manipulation of *A. balhimycina*

Chromosomal integration of genes into the genome of *A. balhimycina* was achieved by direct transformation (D-Trafo), as first described for *Amycolatopsis mediterranei*^63^ and later adapted for *A. balhimycina*.^61^ Demethylated plasmid DNA was isolated from *E. coli* JM110 or ET12567 and used for the transformation of *A. balhimycina* after 48 h of growth. Correct integration was confirmed by PCR.

#### Generation of *A. balhimycina* deletion mutants

In-frame *A. balhimycina* deletion mutants were generated using the non-replicative plasmid pSP1,^61^ which contains homologous flanking regions of ≈1.5 kB upstream and downstream of the target genes *tba* or *bgtfB*, resulting in the plasmids pSP1Δ*tba* and pSP1Δ*bgtfB*, respectively. *A. balhimycina* was transformed with the plasmids using D-Trafo. For single mutants, pSP1Δ*tba* and pSP1Δ*bgtfB* were introduced into *A. balhimycina* wildtype, whereas for the generation of the double mutant *A. balhimycina ΔbhaAΔbgtfB,* we introduced pSP1Δ*bgtfB* into the existing mutant *A. balhimycina ΔbhaA*.^28^ Erythromycin resistant clones harboring either pSP1Δ*tba* or pSP1Δ*bgtfB* were confirmed by PCR using the primers P1-2 (Table S4), and subsequently used for the stress protocol to increase the frequency of double crossover events.^28^ The resulting protoplasts were streaked onto R5 agar plates and R5 agar plates supplemented with 50 μg/mL erythromycin for selection. Erythromycin sensitive clones were screened for in-frame deletion by PCR using primers P9-10 and P15-16 (Table S4).

#### Crude membrane preparation

Crude membranes of *A. balhimycina* were prepared as reported previously by the group.^64^ In brief, 300 mg of culture wet weight was used. The cells were washed with 1xPBS, resuspended in 750 μL buffer K (50 mM triethanolamine (TEA), pH 7.5, 250 mM sucrose, 1 mM EDTA, 1 mM MgCl_2_, 10 μg/mL DNAse, 2 mg/mL lysozyme, 1:100 protease inhibitor cocktail (Sigma-Aldrich (P8849)) and incubated for 30 min at 4°C. Afterward, the cells were mixed with glass beads (Ø = 150–212 μm) and lysed through a bead mill (2 min, continuously). Cell debris was removed by centrifugation at 10,000 x *g* for 10 min. The crude membranes were precipitated by centrifugation at 55,000 x *g* at 4°C for 45 min and resuspended in 75 μL 1xPBS.

#### BN-PAGE, SDS-PAGE, and immunoblotting

Analysis of native transporter complexes was performed as described previously.^65^ Crude membranes were solubilized for 1 h at 4°C using 1% Lauryl Maltose Neopentyl Glycol (LMNG). Non-solubilized materials were separated by centrifugation at 100,000 *x g* at 4°C for 30 min. Fifteen μg of solubilized membrane proteins were subjected to BN-PAGE (NativePAGE 3 to 12%) before transfer to a PVDF membrane. Similarly, for the analysis of denatured proteins, samples were subjected to SDS-PAGE (SERVAGel TG PRiME 8–16%). Membranes were probed with mouse anti-FLAG primary antibody (1:10,000), rabbit anti-BirA (1:5,000), anti-mouse secondary antibody (1:10,000), anti-rabbit secondary antibody (1:10,000), or Streptavidin DyLight 800 (1:10,000). Detection and analysis were performed using a Li-Cor Odyssey system and image Studio (Li-Cor) (v5.2).

#### Balhimycin production, export assay and bioactivity assay

All relevant strains were inoculated in 20 mL of R5 medium and incubated for 48 h as preculture. Five mL of the preculture was used for inoculation of 100 mL main culture in R5 medium. After 96 h of growth, 10 mL of every culture was sampled and separated by centrifugation into supernatant and mycelium. The supernatant was directly used for bioactivity assays as well as for High Performance Liquid Chromatography-Electrospray Ionisation-Mass Spectrometry (HPLC-ESI-MS) measurements. The mycelium was further processed in order to extract intracellular balhimycin. First, it was washed with 10 mL deionized water (diH_2_O), 5 mL carbonate buffer (pH 9.7) and again with 10 mL diH_2_O, to remove trace amounts of cell wall bound balhimycin. Then, the mycelium was lyophilized and weighed for dry cell weight (DCW) determination and used for normalization. Five mL of methanol was used to extract balhimycin from the mycelium (12–16 h). The methanol fraction was separated from the mycelium by centrifugation and collected. The mycelium was washed with 5 mL diH_2_O and the supernatant was also collected. The methanol and water wash fractions were pooled and the extract was evaporated using a Genevac EZ-2 (Genevac Ltd, Suffolk, UK). Finally, the evaporated extracts were dissolved in 0.3 mL of H_2_O and used for bioactivity assays as well as HPLC-ESI-MS measurements. For bioactivity assays we used MM1 medium^66^ indicator plates containing *B. subtilis* DSM10 spores (5x10^6^ spores/mL) and applied 30–40 μL culture supernatant of various *A. balhimycina* strains. The plates were incubated at 37°C overnight.

#### HPLC-MS analysis and quantification of balhimycin

For the detection of balhimycin 2.5 μL of the culture supernatants and mycelium extracts were analyzed by means of HPLC–ESI-MS using a Nucleosil 100-C18 column (3 μm, 100 by 2 mm) (precolumn, 10 by 2 mm) (Dr. Maisch GmbH, Ammerbuch-Entringen, Germany) coupled to an ESI mass spectrometer. LC-MS measurements were obtained from the liquid chromatograph/mass selective detector (LC/MSD) Ultra Trap system XCT 6330 (Agilent Technologies, Waldbronn, Germany). Analysis was carried out at a flow rate of 400 μL/min with gradient elution. Solvent A was 0.1% formic acid, and solvent B 0.06% formic acid in acetonitrile. Gradient elution was performed as follows: t0 = 0% B, t15 = t17 = 100% B, post time 5 min 0% B. The flow rate was 400 μL/min, and the temperature was 40°C. For MS analysis an electrospray ionization (alternating positive and negative ionization) in Ultra Scan mode with a capillary voltage of 3.5 kV and a drying gas temperature of 350°C was used. The detection of m/z values was performed with Agilent DataAnalysis for 6300 series Ion Trap LC/MS 6.1 software (v3.4) (Bruker-Daltonik GmbH).

#### Molecular modeling and molecular dynamics simulation

##### Protein structure prediction

The structural model of relevant transporter members was retrieved from the AlphaFold (v2.2.2) Protein Structure Database using the multimer preset.^26^ All structure models can be found in the Zenodo repository or upon reasonable request. Previous Sav1866 unbiased simulations,^67^ starting from the experimentally available outward open conformation (PDB 2HYD), revealed small conformational changes in the NBD. Alternatively, steered dynamics suggested that, despite the large opening-closing NBD movement, TMD conformational changes are independent. Based on that, we hypothesized that our current simulation length would be insufficient to observe the inward-open transition. This prompted us to generate an inward-open Sav1866 model (UniProt ID: Q99T13, similar to PDB 5MKK), in order to allow a direct comparison against the Tva, Tri and Tba inward-open models. Sav1866 model structure was generated based on the heterodimeric ABC transporter TmrAB (PDB ID: 5MKK), selected based on sequence similarity, using the Homology Model package from Maestro (v2022.4).

##### Binding site prediction and molecular docking

System preparation and docking calculations were performed using the Schrödinger Drug Discovery suite for molecular modeling (v2022.4). Protein−ligand complex was prepared with the Protein Preparation Wizard to fix protonation states of amino acids, add hydrogens, and fix missing side-chain atoms. All ligands for docking were drawn using Maestro and prepared using LigPrep^51^ to generate the 3D conformation, adjust the protonation state to physiological pH (7.4), and calculate the partial atomic charges with the OPLS4 force field. Docking studies with the prepared ligands were performed using Glide (v7.7)^50^^,^^68^ with the flexible modality of induced-fit docking with extra precision (XP), followed by a side-chain minimization step using Prime. Ligands were docked within a grid around 12 Å from the centroid of the predicted binding site pocket, determined using SiteMap.

##### Molecular dynamics simulation

MD simulations for similar ABC transporters were previously validated by the group.^69^^,^^70^ MD simulations were carried out using Desmond,^48^ with the OPLS4 force-field.^49^ The simulated system encompassed the protein-ligand complexes, a predefined water model (TIP3P^71^) as a solvent and counterions. The system was treated in an orthorhombic box with periodic boundary conditions specifying the box’s shape and size as 10 Å distance from the box edges to any atom of the protein. POPC membranes were assigned to the transmembrane helices using the System Setup, with standard options. In all simulations, we used a time step of 1 fs, the short-range coulombic interactions were treated using a cut-off value of 9.0 Å using the short-range method, while the Smooth Particle Mesh Ewald method (PME) handled long-range coulombic interactions.^72^ Initially, the system’s relaxation was performed using Steepest Descent and the limited-memory Broyden-Fletcher-Goldfarb-Shanno algorithms in a hybrid manner, according to the established protocol available in the Desmond standard settings. During the equilibration step, the simulation was performed under the NPT ensemble for 5 ns implementing the Berendsen thermostat and barostat methods.^73^ A constant temperature of 310 K was kept throughout the simulation using the Nose-Hoover thermostat algorithm^74^ and Martyna-Tobias-Klein Barostat^75^ algorithm to maintain 1 atm of pressure, respectively. After minimization and relaxation of the system, we continued with the production step of at least 800 ns, with frames being recorded/saved every 1,000 ps. Five independent replicas were produced for each substrate, resulting in a total of 4 μs simulation/ligand. Trajectories and interaction data are available on the Zenodo repository (see data availability session). The representative structures were selected by inspecting changes in the Root-mean-square deviation (RMSD), meaning for figures a representative frame was selected at random at points of the trajectory where the RMSD for were not fluctuating, after equilibration. Variation of the RMSD values along with the simulation, for both template crystal structures and simulations with docking pose are provided in the repository. Additionally, the changes in the Root-mean-square fluctuation (RMSF), normalized by residue for the protein backbone, are also provided in the Zenodo repository.

#### Transcriptomic analysis

For the transcriptomic analysis, *A. balhimycina* wildtype, Δ*tba* and Δ*bbr* were cultivated in balhimycin production conditions for 48 h followed by RNA extraction using the “Zymo Quick RNA Fungal/Bacterial Kit” (Zymo Research, CN#R2014) according to the manufacturer’s instructions. The RNA integrity was checked by agarose gel electrophoresis and residual DNA was removed by DNAseI treatment using the “DNA-free DNA Removal Kit” (ThermoFischer, CN#AM1906). Successful digestion was initially checked by PCR. The total RNA was quantified with a “Qubit RNA BR Assay Kit” (Thermo Fisher) and RNA integrity was checked by an Agilent 2100 BioAnalyzer with the “RNA 6000 Pico kit” (Agilent). Library preparation and ligation were performed using “Illumina Stranded Total RNA Prep” and “Ribo-Zero Plus Microbiome”, respectively, according to the manufacturer’s instructions. In brief, 100 ng of total RNA per sample were subjected to rRNA depletion, followed by cDNA library construction, adapter ligation, and 15 cycles of barcoding PCR. The obtained libraries were quantified with the “Qubit 1x DNA HS Assay Kit” (Thermo Fisher) and the fragment distribution was checked with an Agilent 2100 BioAnalyzer using the “High Sensitivity DNA Kit” (Agilent). Libraries were subsequently pooled and sequenced on an Illumina NovaSeq 6000 device using “NovaSeq 6000 SP Reagent Kit” (v1.5) (100 cycles) with a run mode 75,10,10,0. The average number of reads obtained was 13-21x106. The sequencing was demultiplexed with the latest version of the nextflow pipeline: nf-core/demultiplex. For demultiplexing, “bcl2fastq” was used and the quality was checked with “fastp”.

Sequencing statistics, including the quality per base and adapter content assessment, were conducted with FastQC (v0.11.8).^76^ All reads mappings were performed against the assembled strain *A. balhimycina* DSM 44591. The strain was annotated using BAKTA (v1.9.1).^77^ The annotation of the cluster genes (positions 6028489 to 6094273) was manually curated. The mappings of all samples were conducted with HISAT2 (v2.1.0),^78^ using the following parameters: spliced alignment of reads was disabled and strand-specific information was set to reverse complemented (HISAT2 parameter --no-spliced-alignment and --rna-strandness "R"). The resulting mapping files in SAM format were converted to BAM format using SAMtools (v1.9).^79^ Mapping statistics, including strand specificity estimation and percentage of mapped reads, were conducted with the RNA-Seq module of QualiMap2 (v2.2.2-a).^80^ Gene counts for all samples were computed with featureCounts (v1.6.4),^81^ where the selected feature type was set to transcript records (featureCounts parameter -t transcript). A quality check for ribosomal rRNA was performed with a self-written script based on the absolute counts of annotated rRNAs. To assess variability across the replicates of each time series, a principal component analysis (PCA) was conducted with the DESeq2 package (v1.28.1).^82^

For the computation of genes differentially expressed between the mutants, DESeq2 was applied to the absolute gene counts as computed with featureCounts. For differences between the two mutants and the wildtype strain, genes with an adjusted *p*-value (FDR) < 0.05 and absolute log2 fold change (FC) > 1 were reported as differentially expressed.

#### Measurement of amino acid levels by metabolomic analysis

For metabolomic measurements, *A. balhimycina* wildtype and Δ*tba* were cultivated under balhimycin production conditions and harvested after 48 h. Two hundred μl of liquid cultures were filtered (Merck, Durapore 0.45 μm PVDF) using a vacuum pump. The obtained biomass was extracted with 1 mL of an acetonitrile:methanol:water solution (40:40:20) for 1 h at −20°C. The mixture was transferred to a tube with glass beads (Ø = 0.1–0.11 mm) and homogenized using a Precellys (2 × 30 s, 6.5 m/s). The mixture was centrifuged at −9°C and 13,000 rpm for 15 min. The supernatant was subsequently used for the LC-MS/MS measurement. Amino acids were measured via targeted LC-MS/MS as described previously.^83^ The obtained ratios were further processed by normalizing the values to the dry cell weight (DCW). These values were then used to calculate the differences between the Δ*tba* mutant and the wildtype. The differences were expressed as log2 fold changes in relation to a 13C internal standard.

#### Proximity dependent biotinylation and proteomic analysis

For the proximity dependent biotinylation studies, *A. balhimycina* was cultivated under balhimycin producing conditions, as described in previous sections. Fifty mL of culture was harvested after 48 h at 5,000 *x g*, 4°C for 10 min. The cell pellet was washed with 20 mL of 1xPBS buffer and centrifuged at 4,600 *x g*, 4°C for 10 min. Biotin labeling was carried out in 20 mL 1xPBS supplemented with 500 μM biotin for 1 h at 37 °C at 650 rpm. The reaction was stopped at 4°C and three subsequent wash steps with ice-cold 1xPBS. The cells were resuspended in 15 mL lysis buffer (0.1 M Tris-HCl (pH 8.0), 0.15 M NaCl, 1 mM EDTA, 10 μg/mL DNAse, 2 mg/mL lysozyme, 1:100 protease inhibitor cocktail (Sigma-Aldrich (P8849), 1 mM MgCl_2_) and sequentially disrupted by sonication (Branson sonifier 250) (output control "4", 35% duty cycle 2 × 30 s/10 s pause), followed by homogenization by a Constant System CF-1 homogenizer (I&L Biosystems) (2x 40,000 psi). The cell lysate was cleared twice at 10,000 *x g*, 4°C for 3 and 10 min. The protein concentration was measured using Pierce BCA Protein Assay Kits (Thermo Fisher). Five mg of total protein (Input) was used for enrichment of biotinylated proteins using 150 μL of MagStrep Strep-Tactin beads (IBA). Binding of proteins to the streptavidin magnetic beads was performed by overhead rotation at 4°C overnight. Washing and elution were performed as described by the manufacturer. Input, flowthrough, and elution fractions were applied to an SDS-PAGE gel as a control and probed after western blotting with an anti-BirA antibody or streptavidin.

For subsequent mass spectrometry analysis, 20 μL of every eluate fraction was loaded on an SDS-PAGE gel and run until the loading front reached approx. 1 cm of the gel. The proteins were stained using a standard Coomassie staining solution. Until further processing, the gel was stored in 5% acetic acid. The bands were excised and cut further into small pieces. Proteins were then digested in the gel with trypsin, whereby all incubation steps were carried out under shaking conditions: for destaining, gel pieces were washed three times with 5 mM ammonium bicarbonate (ABC) in acetonitrile (ACN) (1:1, v/v) for 20 min. After a dehydration step with 100% ACN for 10 min, disulfide bonds were reduced by adding 10 mM dithiothreitol (DTT) in 20 mM ABC for 45 min at 56°C. Thiol groups were carbamidomethylated with 55 mM iodoacetamide (IAA) in 20 mM ABC for 45 min in the dark. Subsequently, gel pieces were washed two times with 5 mM ABC in ACN (1:1, v/v) for 20 min and dehydrated with 100% ACN for 15 min. Liquid was evaporated by vacuum centrifugation for 10 min and sequencing grade trypsin (Promega) was added in a concentration of 12.5 ng/μL in 20 mM ABC, pH 8.0. Gel pieces were soaked for 10 min at room temperature (RT), then covered with 20 mM ABC, and proteins were digested at 37°C overnight. Peptide extraction was performed in three consecutive steps with different extraction buffers for 30 min: first 3% (v/v) trifluoroacetic acid (TFA) in 30% (v/v) ACN was added, followed by 0.5% (v/v) formic acid (FA) in 80% (v/v) ACN, and completed by 100% ACN. Supernatants were pooled and ACN was evaporated by vacuum centrifugation. Peptides were further purified with C18 StageTips^84^ and analyzed on an EASY-nLC 1200 UHPLC coupled to a Q Exactive HF mass spectrometer (both Thermo Scientific) as described previously^85^ with slight modification: peptides were eluted from the analytical column using a 46 min segmented gradient of 10-33-50% of HPLC solvent B (80% acetonitrile in 0.1% formic acid) at a flow rate of 200 nL/min. In the mass spectrometer, MS and MS/MS spectra were acquired at resolution 60k. Full MS target value and maximum IT were set to 3x10^6^ and 25 ms, respectively. In each scan cycle, the 7 most intense precursor ions were picked up, whereby MS/MS target value was set to 10^5^ charges with a maximum IT of 110 ms.

MS data were processed using default parameters of the MaxQuant software (v2.4.12.0).^86^ Extracted peak lists were submitted to database search using the Andromeda search engine^87^ to query a target-decoy^88^ database of *A. balhimycina* (9,427 entries, downloaded on 30^th^ of January 2024) and 286 commonly observed contaminants.

In database search, full tryptic specificity was required and up to two missed cleavages were allowed. Carbamidomethylation of cysteine was set as fixed modification, whereas protein N-terminal acetylation, and oxidation of methionine were set as variable modifications. For the main search, the peptide mass tolerance was set to 4.5 ppm, and 20 ppm at the fragment ion level. Peptide, protein, and modification site identifications were filtered at a false discovery rate (FDR) of 0.01. The iBAQ (Intensity Based Absolute Quantification) and LFQ (Label-Free Quantification) algorithms were enabled, as was the “match between runs” option.^89^^,^^90^

Downstream statistical analysis was performed using LFQ values and according to Aguilan et al.^91^ with an additional filtering step to remove hits that were only detected in one replicate and condition.

### Quantification and statistical analysis

The extra- and intracellular amounts of balhimycin were quantified using the HPLC-MS data. For this purpose, the peak intensities of each data point in the MS chromatogram corresponding to the masses of balhimycin were summed to a total intensity value. The concentration of balhimycin in the analyzed samples was calculated by using a standard curve, in which the total intensities of pure balhimycin were used. The concentration values were used to determine the total amount of balhimycin in the supernatant or mycelium extracts, respectively. The export and accumulation levels of balhimycin were normalized to the dry cell weight (DCW). Normalized values were used for statistical analysis using R (v4.3.1)^92^ including the additional packages ”dplyr” (v1.1.3),^93^ “readxl” (v1.4.3),^94^ “ggplot2” (v3.5.0),^95^ and “reshape” (v.1.4.4).^96^ We used the “Wilcoxon signed-rank test” and the “Benjamini-Hochberg Procedure” to calculate significance levels (*p*-values) for all data analyzed. *p*-values <0.05 were considered as statistically significant. The results of this analysis are found in Figures 2 and 4 and the respective figure legend. Every datapoint represent one independent experiment and quantification (n). At least three independent experiments were performed per strain. Figure 2: *A. balhimycina* wildtype (*n* = 11), Δ*tba* (*n* = 12), Δ*tba* + *tba* (*n* = 12), Δ*tba* + *tba*^*3xFLAG*^ (*n* = 11), Δ*tba* + *tri*^*3xFLAG*^ (*n* = 11), Δ*tba* + *tva*^*3xFLAG*^ (*n* = 8), Δ*tba* + *tbaE545Q*^*3xFLAG*^ (*n* = 6), Δ*tba* + *sav1866*^*3xFLAG*^ (*n* = 8). Figure 4: *A. balhimycina* wildtype (*n* = 3), Δ*tba* (*n* = 3), Δ*tba* + *tba*^*3xFLAG*^ (*n* = 3), Δ*tba* + *tbaQ309G*^*3xFLAG*^ (*n* = 6), Δ*tba* + *tbaR310A*^*3xFLAG*^ (*n* = 6), Δ*tba* + *tbaT316A*^*3xFLAG*^ (*n* = 6).

## Supplemental information

Document S1. Figures S1–S12 and Tables S3–S6

Table S1. List of used transporters with supporting information

Table S2. Statistical analysis of quantitative measurements
