# Supplementary material for: Unveiling the substrate specificity of the ABC transporter Tba and its role in glycopeptide biosynthesis
Source: iScience. 2025 Mar 3;28(4):112135. doi: 10.1016/j.isci.2025.112135 (PMC11960670; doi:10.1016/j.isci.2025.112135)
Supplement: Document S1. Figures S1–S12 and Tables S3–S6 [file mmc1.pdf]

iScience, Volume *28*

Supplemental information

Unveiling the substrate specificity of the ABC transporter Tba and its role in glycopeptide biosynthesis

Nicola Gericke, Dardan Beqaj, Thales Kronenberger, Andreas Kulik, Athina Gavriilidou, Mirita Franz-Wachtel, Ulrich Schoppmeier, Theresa Harbig, Johanna Rapp, Iwan Grin, Nadine Ziemert, Hannes Link, Kay Nieselt, Boris Macek, Wolfgang Wohlleben, Evi Stegmann, and Samuel Wagner

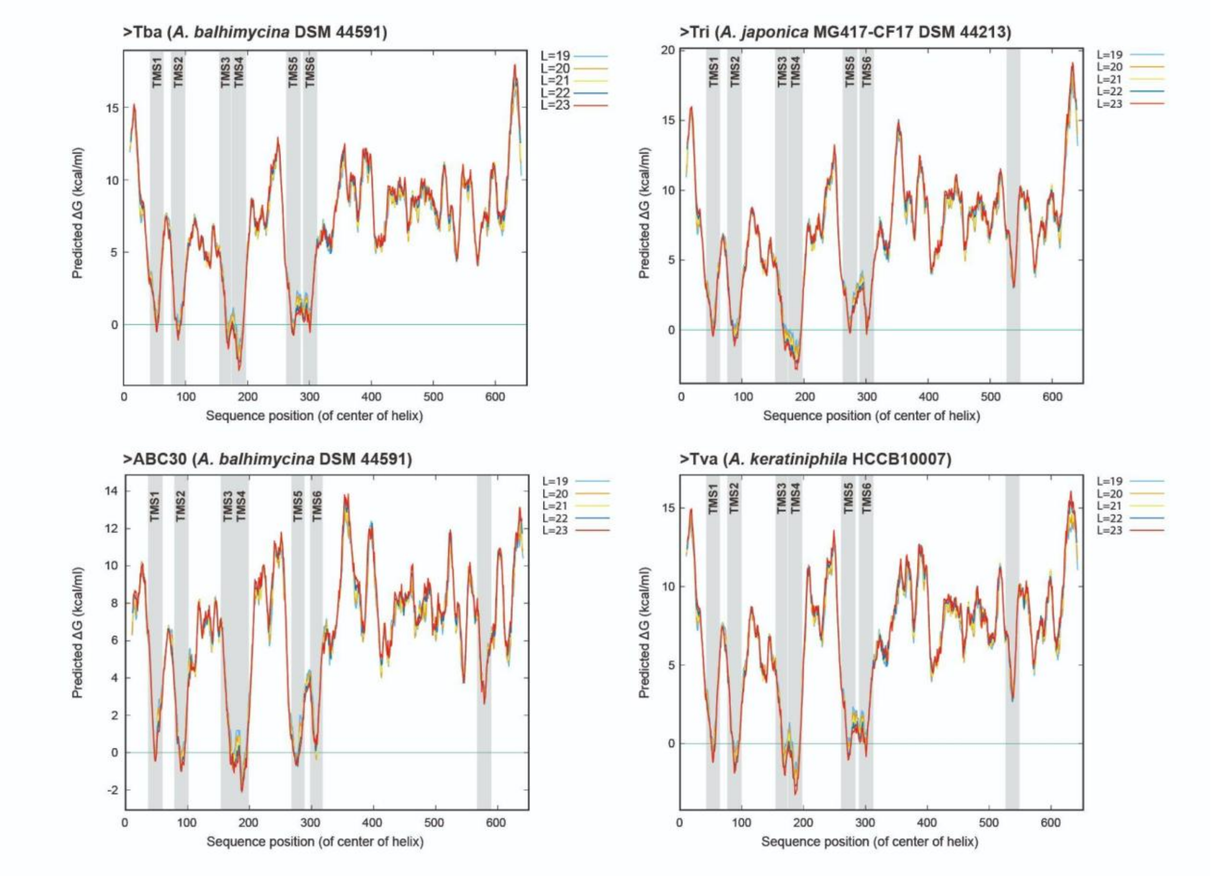

Figure S1. Prediction of transmembrane topology of different GPA ABC transporters. Prediction of transmembrane topology of ABC transporters encoded in the GPA BGC of *A. balhimycina* (Balhimycin producer), *A. japonica* (Ristomycin producer), *A. keratiniphila* (Vancomycin producer), and the non GPA transporter Abc30 of *A. balhimycina*. Topology was predicted based on the membrane integration propensity of segments (19-23 AA)[1]. Putative transmembrane segments (TMS) are marked in gray.

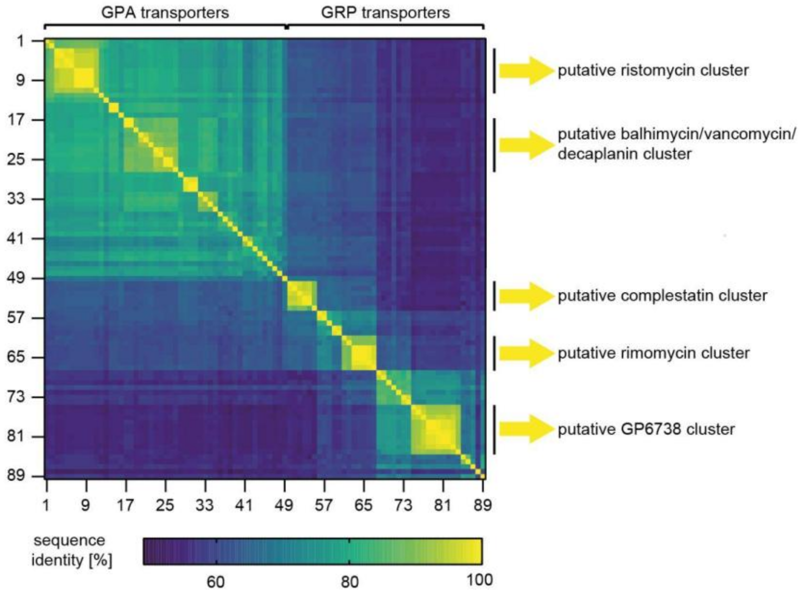

Figure S2. Similarity matrix of amino acid sequence identities of transporters. Heatmap representation of amino acid sequence identities of all 89 GPA and GRP associated ABC transporters. Identities were determined by a BlastP pairwise comparison analysis and are displayed in percent (%) of identity. Each number represents one transporter. The corresponding strains from which the sequences originate and numberings used in the heatmap are listed in supplementary table 1.

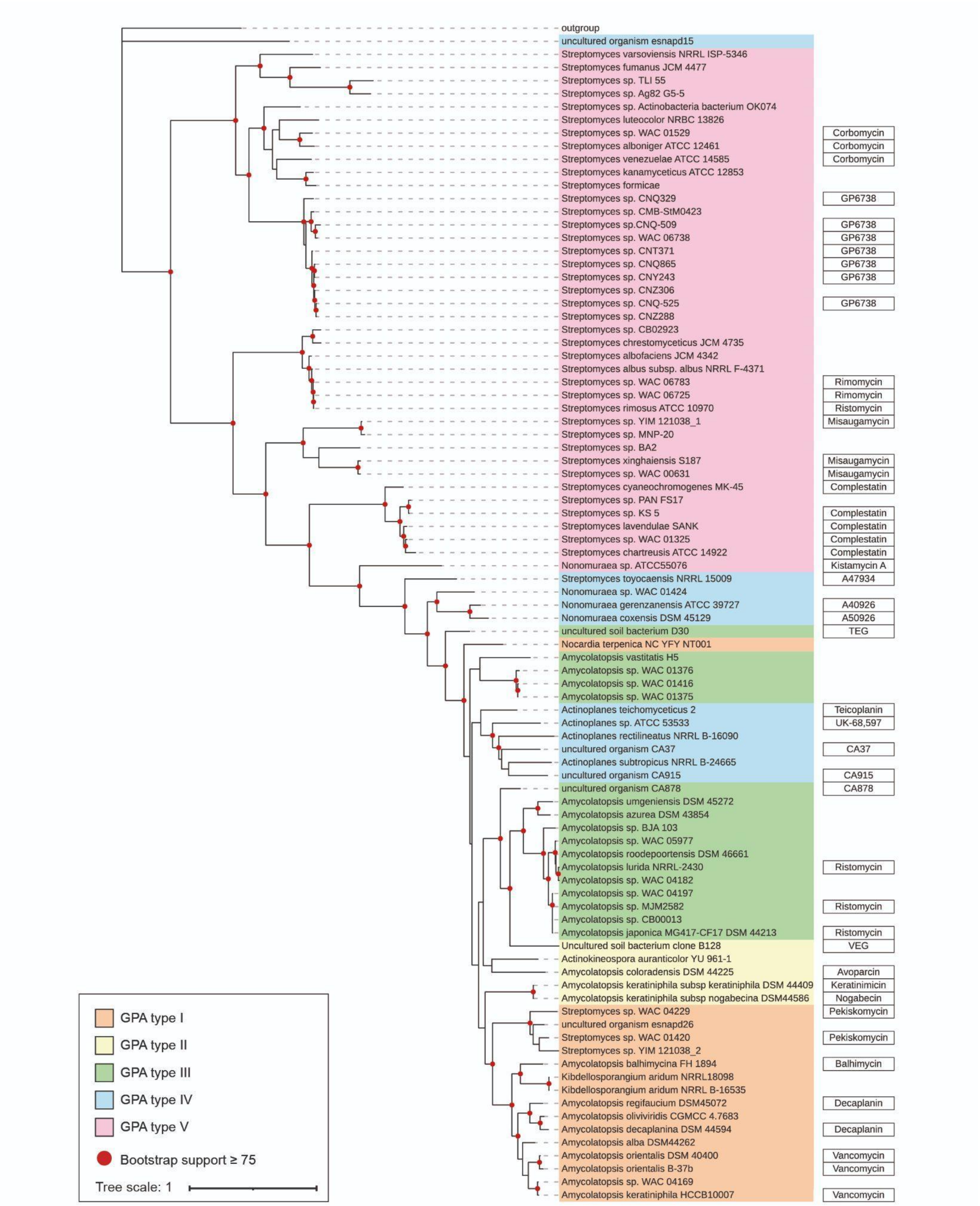

Figure S3. Evolutionary relationship within the group of GPA and GRP associated ABC exporters. Phylogenetic tree of ABC transporters encoded in BGC of GPAs and GRPs. The tree was calculated using the Maximum Likelihood (LG+F+G) algorithm with 1000 bootstrap repetitions. Nodes with bootstrap support ≥ 75 are marked by red dots. The tree was rooted using a putative ABC transporter encoded in an unknown BGC in *A. balhimycina* (Abc30). The strain from which the cluster and thus the associated ABC transporter originate is indicated in the tree. (Predicted) GPA types are labeled accordingly: type I (orange), type II (yellow), type III (green), type IV (blue), type V (pink). Known and predicted GPAs are mentioned behind the branches.

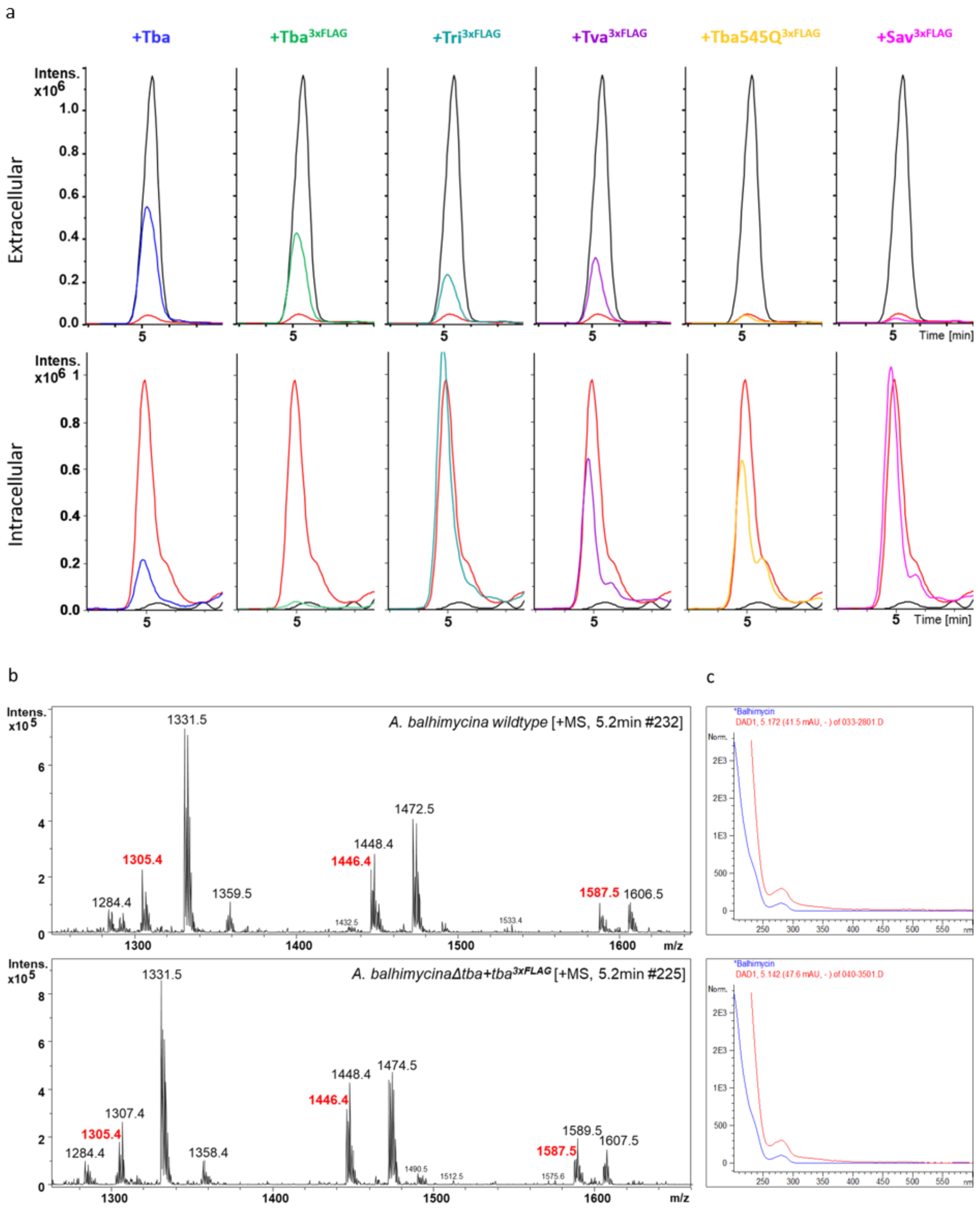

Figure S4. Export assay of balhimycin by diverse ABC transporters. a: HPLC-MS chromatograms of the culture supernatants (Extracellular) as well as mycelium extracts (Intracellular). The peaks (retention time 5-5.5 min) represent the extracted ion chromatogram (EIC) of the protonated balhimycin mass *m/z* 1446.41 $[M+H]^+$ (positive mode, smoothed 10.63-10.76 GA). In each chromatogram view, the EIC of *A. balhimycina* wildtype (black) and Δ*tba* (red) are overlaid with the respective EIC of the Δ*tba* mutant complemented with different transporters. **b:** Depicted mass spectra of *A. balhimycina* wildtype (top) and *A. balhimycina Δtba+tba*^{3xFLAG}(bottom*)* showing detected masses of the balhimycin molecules as $[M+H]^+$(red). **c:** UV-spectra of balhimycin derived from HPLC-DAD analysis of supernatants of *A.*

balhimycina wildtype (top) and *A. balhimycinaΔtba+tba*^{3xFLAG} (bottom) compared to in-house UV database of balhimycin standard.

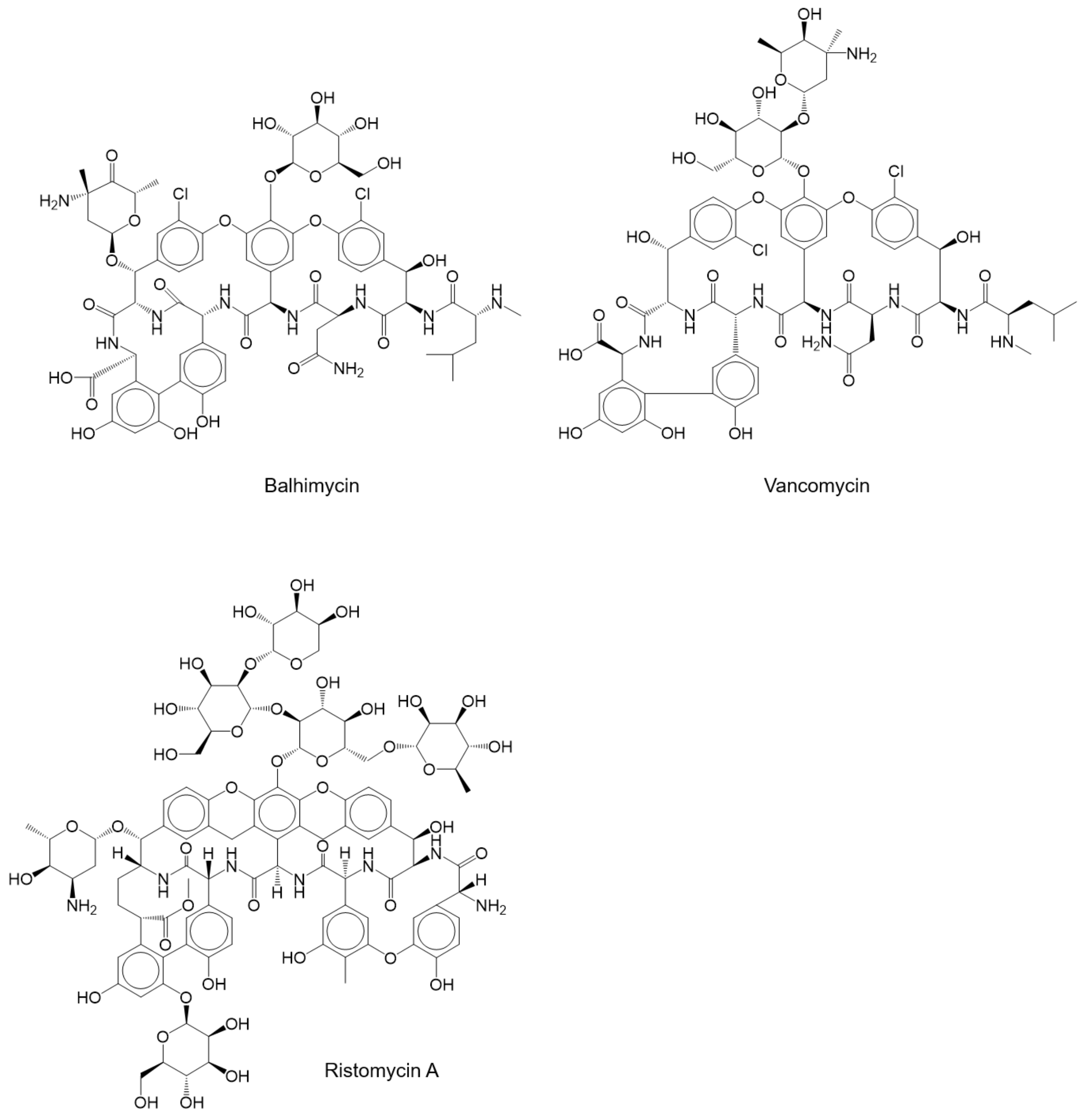

Figure S5. Chemical structure of the glycopeptides molecules.
Balhimycin (ChemSpider ID10249894), vancomycin (ChemSpider ID14253) and ristomycin A (ChemSpider ID16736169)

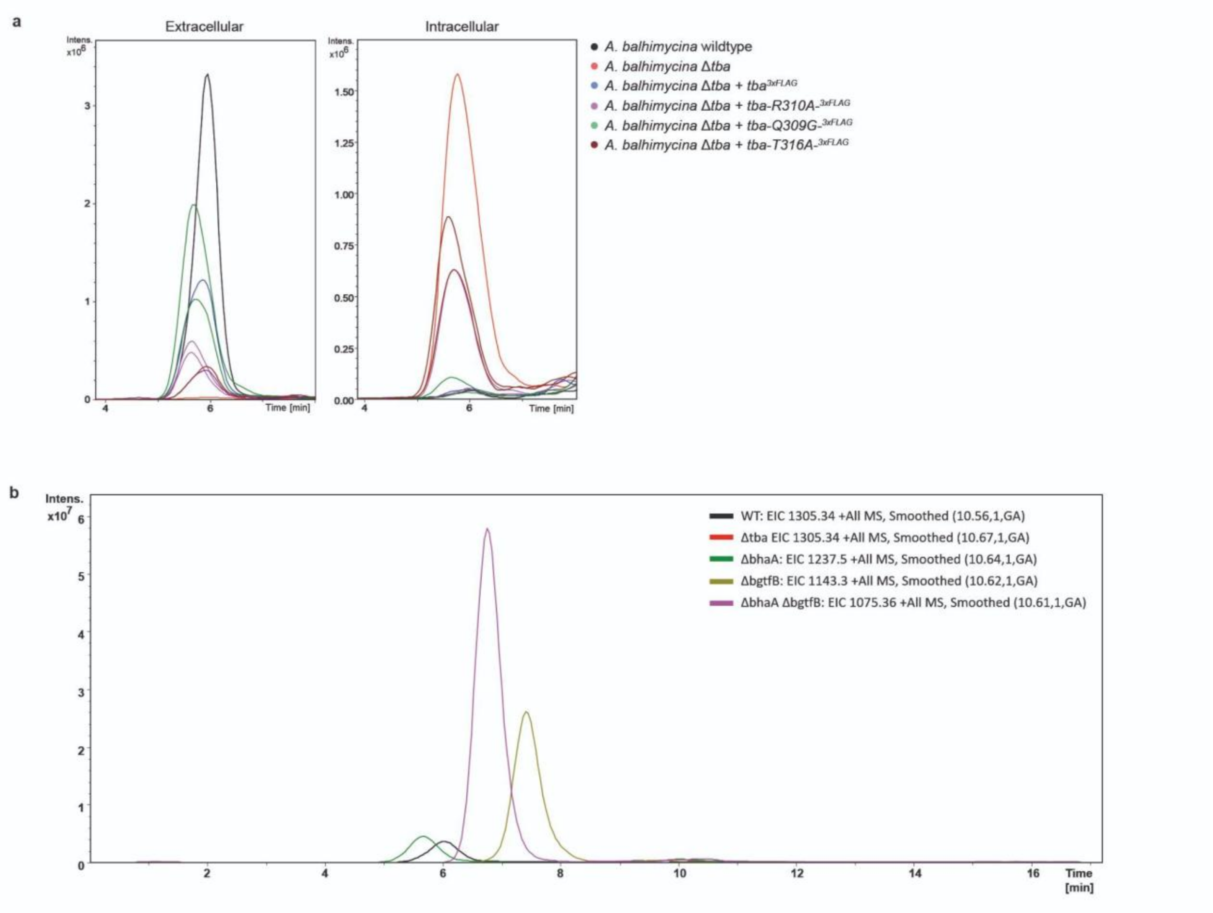

Figure S6. HPLC-MS chromatograms of mutants carrying point mutations in the TMD and mutants lacking the genes for chlorination and glycosylation. a: HPLC-MS chromatograms of the culture supernatants (Extracellular) as well as mycelium extracts (intracellular) of the mutants expressing *tba-Q309G-*^{3xFLAG}, *tba-R310A-*^{3xFLAG} and *tba-T316A-*^{3xFLAG}. The peaks (retention time 5-5,5 min) represent the extracted ion chromatogram (EIC) of the protonated balhimycin mass 1446.41 m/z [M+H] (positive mode, smoothed 10.63-10.76 GA). The EICs of each mutant are overlaid and compared to the wildtype (black) and Δ*tba* (orange) as well as *tba*^{3xFLAG}. **b:** HPLC-MS analyzed culture supernatants of mutants, missing the *bhaA* and *bgtfB* genes. Each chromatogram represents EICs of the corresponding derivative produced by the mutant. As controls, the EICs of wildtype (black) and Δ*tba* (red) are overlaid.

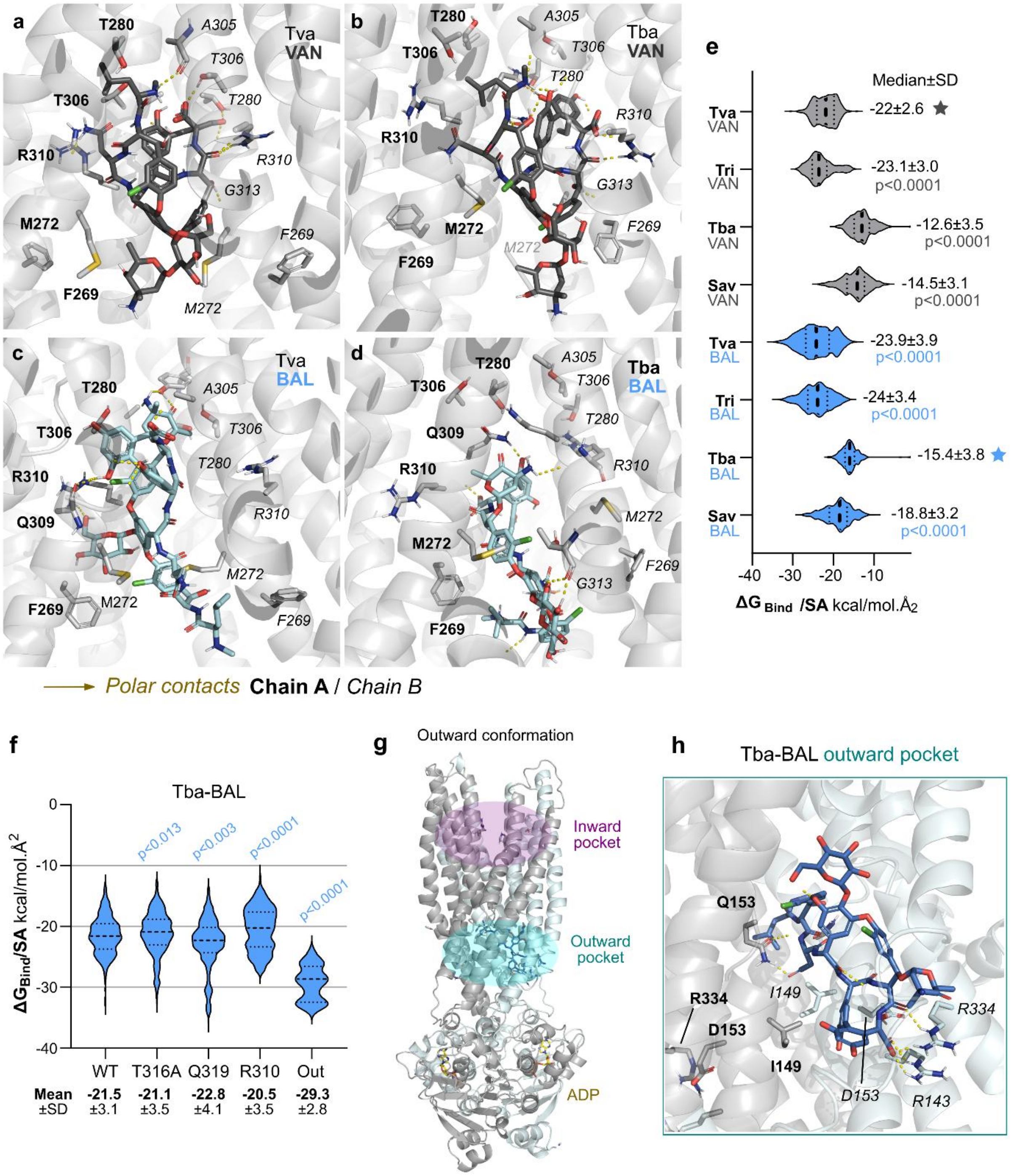

Figure S7. GPA potential binding mode and their respective predicted binding energies. Representative conformation from the MD simulation for the vancomycin (**VAN**) binding transporters Tva (**a**) and Tba (**b**), as well as their balhimycin (**BAL**) counterparts in (**c, d**). Conformations highlight that GPAs proposed binding mode points the R1-sugar moieties towards the cytoplasmic cavity beyond the substrate binding pocket (in the TMD). Predicted binding energy (**e, f**). Violin plot depicting the variation of free binding energy calculated along the trajectory normalized by the ligand's surface for: (**E**) WT-GPA transporters with VAN (gray) or BAL (blue) and (**f**) for different point mutations of Tba bound to BAL. Median energy values are depicted above each ligand. Mann-Whitney tests were performed to compare all groups against the control group's cumulative distribution, highlighted with a star from the respective group; exact p-values are depicted when available. Comparisons between the same transporter with different binding GPA yielded no significant results and therefore are not

displayed. **g**: 3D representation of a type IV GPA transporter Tba based on the SAV1866 (PDB 2HYD) followed by short MD simulations (5x200 ns). **h:** Proposed binding mode for relevant GPAs, generated from representative conformations from the MD simulation for the transporter Tba bound to balhimycin.

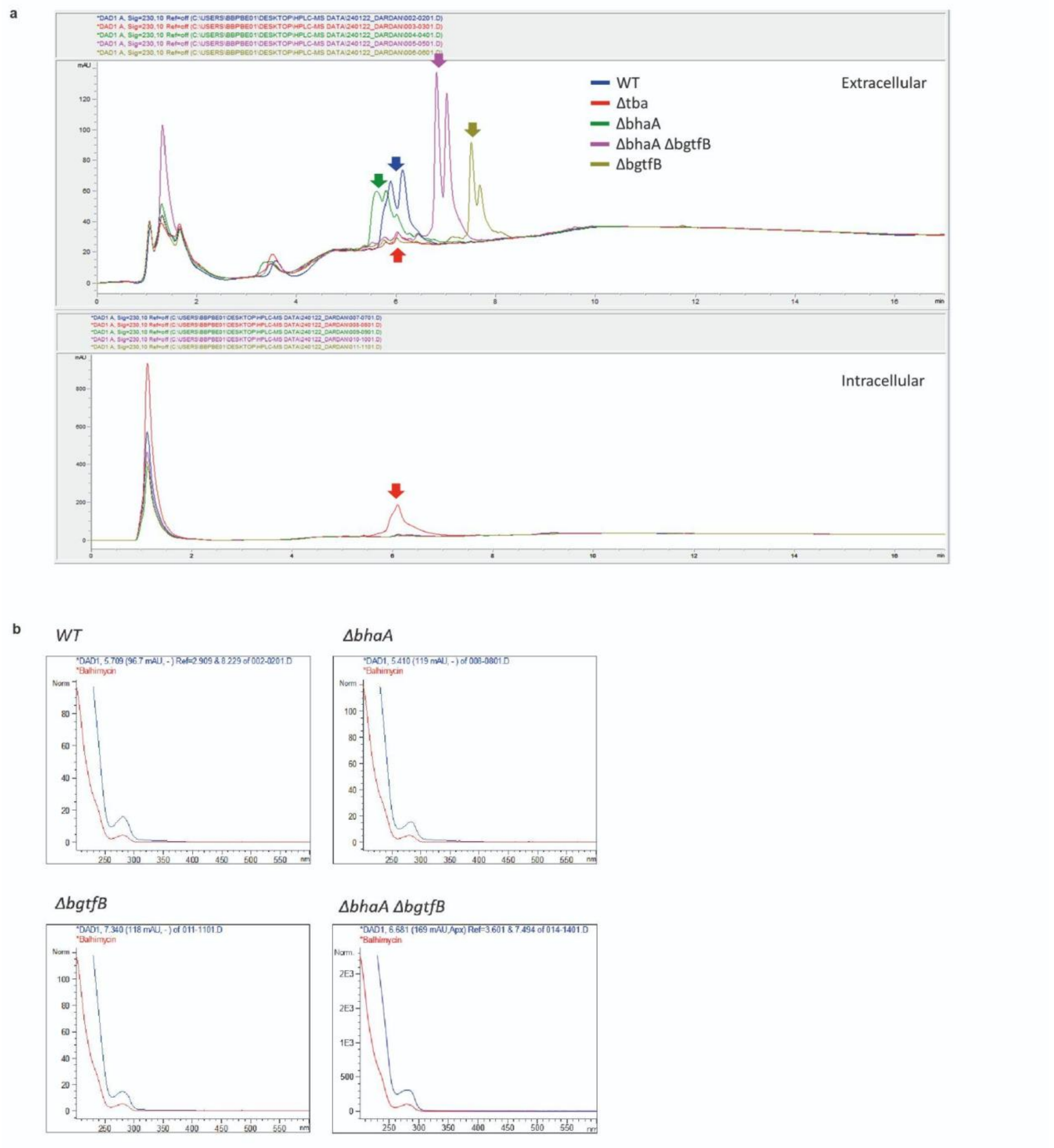

Figure S8. UV spectra of the analyzed deletion mutants Δ*bhaA*, Δ*bgtfB* and Δ*bhaA*Δ*bgtfB*. a: The UV spectrum at 230 nm after HPLC-DAD of each analyzed supernatant are overlaid and marked with arrows indicating the peak corresponding to the balhimycin derivatives produced by the respective mutant. **b:** UV-spectra of balhimycin and its derivatives derived from HPLC-DAD analysis of supernatants of each *A. balhimycina* mutant (blue) compared to in-house UV database of balhimycin standard (red).

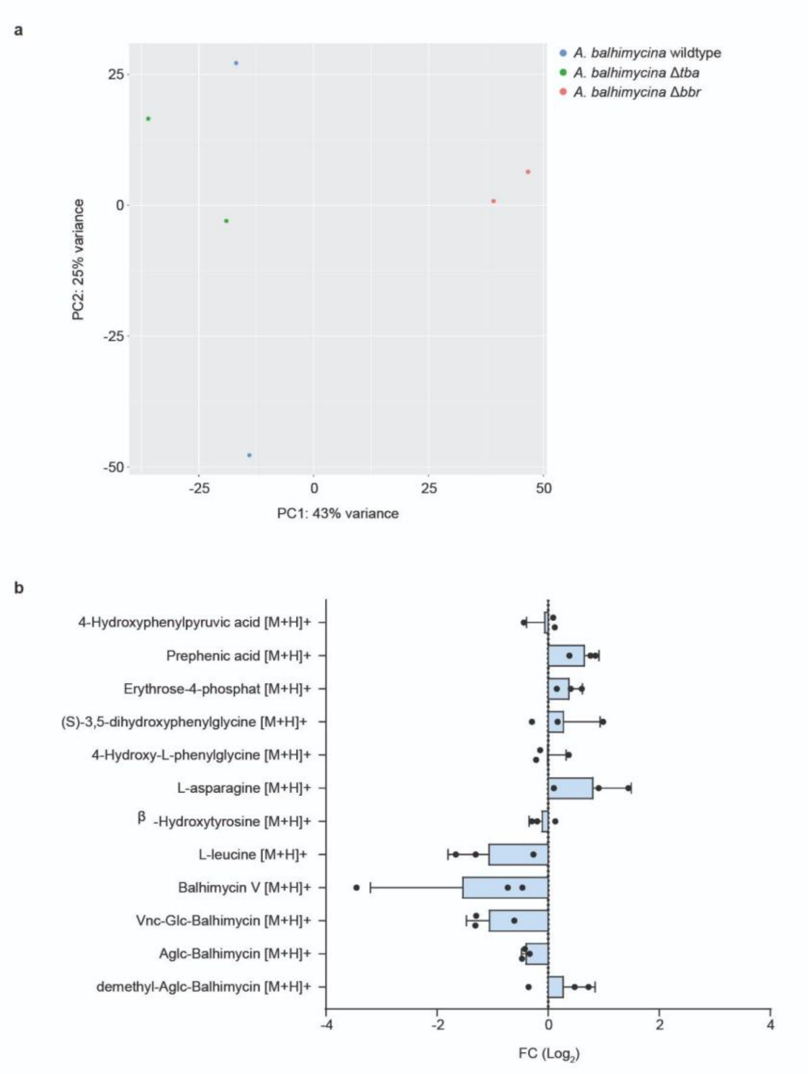

Figure S9. Transcriptome and metabolome analysis of *A. balhimycina* Δ*tba*. a: Comparison of two individual replicates of *A. balhimycina* wildtype (blue), Δ*tba* (green), and Δ*bbr* (salmon) using principal component analysis (PCA). **b:** Levels of balhimycin intermediates after 48 h measured by flow-injection mass spectrometry. Values display *A. balhimycina* Δ*tba* in comparison to *A. balhimycina* wildtype**.**

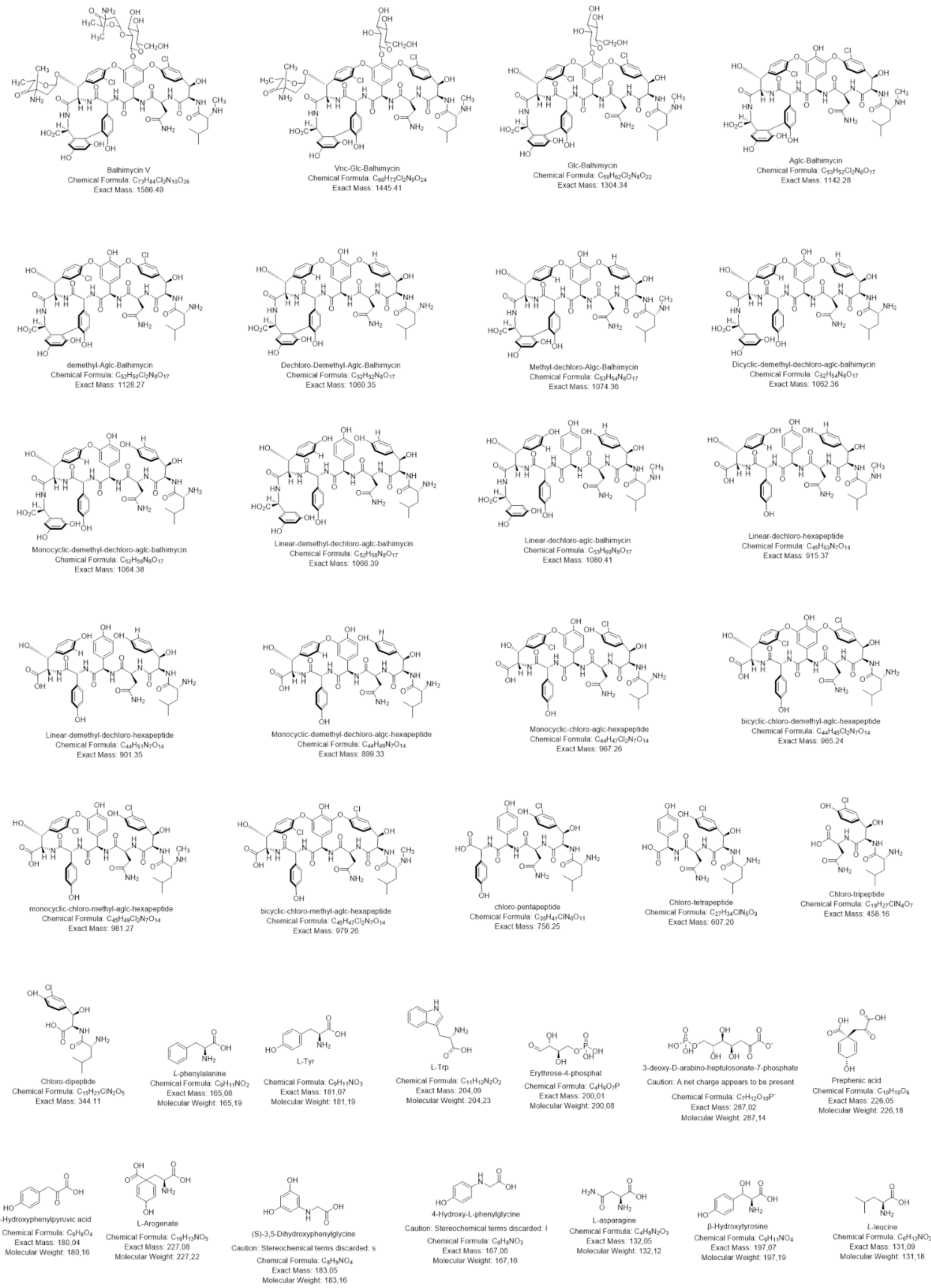

Figure S10. List of balhimycin intermediates. Structure, chemical formula, exact mass and molecular weight of balhimycin, putative intermediates, and precursors.

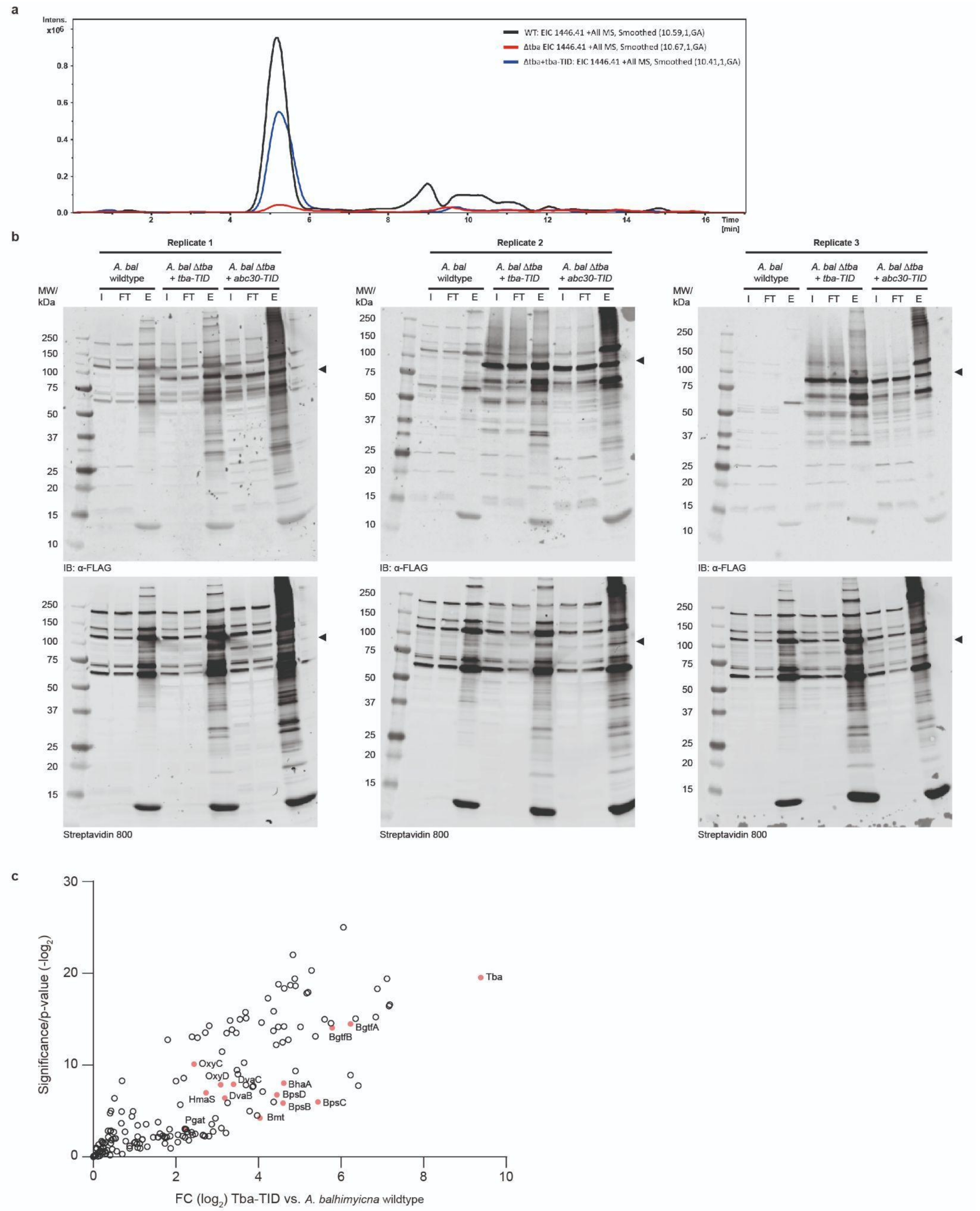

Figure S11. Proximity dependent biotinylation. a: HPLC-MS chromatograms of the culture supernatants of *A. balhimycina* Δ*tba* complemented with *tba-TID*. The peaks (retention time 5-5.5 min) represent the extracted ion chromatogram (EIC) of the protonated balhimycin mass *m/z* 1446.41 $[M+H]^+$ (positive mode, smoothed 10.41-10.67 GA). **b:** Immunoblotting (top) and fluorescence labeling with Streptavidin 800 (bottom) after SDS-PAGE of input (I) biotinylated proteins after enrichment (E) and flowthrough after enrichment (FT) from the recombinant strains expressing TID fusion proteins. **c:** Representation of all log2 fold change positive proteins detected by LC-MS/MS after biotinylation. Hits of Tba-TID compared to the wildtype strain *A. balhimycin*a without TID.

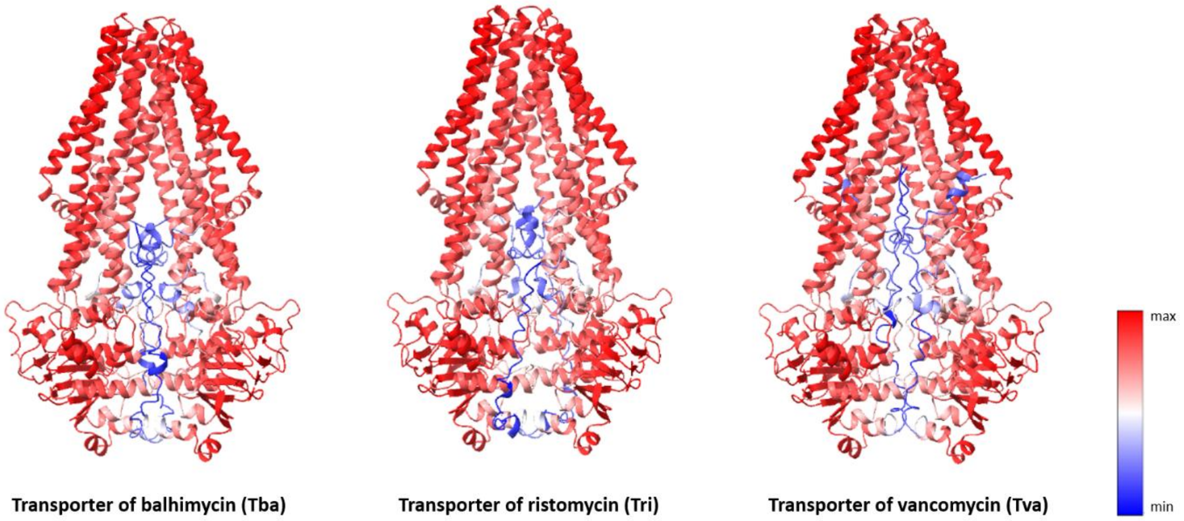

Figure S12. AlphaFold2 models of Tba, Tri and Tva and coloration based on B-factor coloured by model per-residue confidential score. The coloration is displayed in red as maximum and in blue as lowest value of the per-residue score. TMD regions were modelled with high confidence while the N-terminal could not, therefore it was removed from the calculations, to generate a truncated protein model.

Table S3. Mass list of balhimycin intermediates and precursors

Nr	Name	Molecular formula	Exact Mass
1	Chloro-dipeptide	C15H21ClN2O5	344.113901
2	Chloro-tripeptide	C19H27ClN4O7	458.156829
3	Chloro-tetrapeptide	C27H34ClN5O9	607.204508
4	chloro-pentapeptide	C35H41ClN6O11	756.252187
5	bicyclic-chloro-methyl-aglc-hexapeptide	C45H47Cl2N7O14	979.255809
6	monocyclic-chloro-methyl-aglc-hexapeptide	C45H49Cl2N7O14	981.271459
7	bicyclic-chloro-demethyl-aglc-hexapeptide	C44H45Cl2N7O14	965.240159
8	Monocyclic-chloro-aglc-hexapeptide	C44H47Cl2N7O14	967.255809
9	Monocyclic-demethyl-dechloro-algc-hexapeptide	C44H49N7O14	899.333753
10	Linear-demethyl-dechloro-hexapeptide	C44H51N7O14	901.349402
11	Linear-dechloro-hexapeptide	C45H53N7O14	915.365053
12	Linear-dechloro-aglc-balhimycin	C53H60N8O17	1080.407647
13	Linear-demethyl-dechloro-aglc-balhimycin	C52H58N8O17	1066.391997
14	Monocyclic-demethyl-dechloro-aglc-balhimycin	C52H56N8O17	1064.376347
15	Dicyclic-demethyl-dechloro-aglc-balhimycin	C52H54N8O17	1062.360697
16	Methyl-dechloro-Algc-Balhimycin	C53H54N8O17	1074.360697
17	Dechloro-Demethyl-Aglc-Balhimycin	C52H52N8O17	1060.345047
18	demethyl-Aglc-Balhimycin	C52H50Cl2N8O17	1128.267103

19	Aglc-Balhimycin	C53H52Cl2N8O17	1142.282753
20	Glc-Balhimycin	C59H62Cl2N8O22	1304.335578
21	Vnc-Glc-Balhimycin	C66H73Cl2N9O24	1445.414557
22	Balhimycin V	C73H84Cl2N10O26	1586.493536
Precursors			
NR	Name	Molecular formula	Exact mass
1	L-leucine	C6H13NO2	131.094629
2	β-Hydroxytyrosine	C9H11NO4	197.068809
3	L-asparagine	C4H8N2O3	132.053493
4	4-Hydroxy-L-phenylglycine	C8H9NO3	167.058244
5	(S)-3,5-dihydroxyphenylglycine	C8H9NO4	183.053159
6	Erythrose-4-phosphat	C4H9O7P	200.008593
7	3-deoxy-D-arabino-heptulosonate-7-phosphate	C7H12O10P	287.016812
8	Prephenic acid	C10H10O6	226.04774
9	4-Hydroxyphenylpyruvic acid	C9H8O4	180.04226

Table S4 | Primers used in this study

ID	Primer	Sequence (5’□3’)
P1	seq_ermE_prom_f	TTG TGG GCA CAA TCG TGC CGG
P2	seq_lacZa_r	GCA CTG GCC GTC GTT TTA CAA CGT
P3	tba_UP_f	TAA AGG GAG AGA CGA ATT CGA GCT CGG TAC GCC AAG CCG CAC CCG C
P4	tba_UP_r	GCT GCG GAA TTC ATC CAT CCG GTC ACC TCT CT
P5	tba_Down_f	AGA GGT GAC CGG ATG GAT GAA TTC CGC AGC GCG GAC CA
P6	tba_Down_r	GCA GGT CGA CTC TAG AGG ATC CCC GGG TAC GCT CGG TGC CCG CCC
P7	tba_pRM4_f	CTG CAG GAA TTC GAT ATC AAG CTT TCA TCC TCC GTA GCC CAT GTG
P8	tba_pRM4_r	CCA GGG GAG GAC CCA TAT GAT GGA CAT GGT GTT GCG TTT
P9	KO_tba_f	ACG CCC CGC ACG TGG TGG CGT
P10	KO_tba_r	TCG GAC GCG CCC TCC TCG GCG GCG
P11	bgtfB_down_f	GCG ATA TCC GCG AAA CTG CTG CTC G
P12	bgtfB_down_r	GGA TCG TCT AGA TGG CCA CCT TCG C
P13	bgtfB_UP_f	GTG GAA TTC GGC AGC TCG TCC GGA C
P14	bgtfB_UP_r	TTG ATA TCC GGT TGT TCC GCT CCC
P15	KO_bgtfB_f	AAC CTA CGG AAC AGA GGG TGC
P16	KO_bgtfB_r	GTC TCC TCT TTG CTT TGT CTT CGA AGA
P11	gib_uni_pRM4_f	TCT AGA GTC GAC CTG CAG CCC GAG
P12	gib_uni_tba_r	TCC TCC GTA GCC CAT GTG TTG GAT C
P13	gib_pRM4_3xFLAG_f	GAT CCA ACA CAT GGG CTA CGG AGG ATC TAG AGA CTA CAA AGA CCA TGA C

P14	gib_pRM4_3xFLAG_r	CTC GGG CTG CAG GTC GAC TCT AGA TCA TTT GTC ATC GTC ATC CTT GTA ATC
P15	gib_pRM4_Tri2_f	ATA AGC TAG CCA GGG GAG GAC CCA TAT GGA AGT AAT GTT GCG CTT CGG
P16	gib_pRM4_Tri_r	GCT CGG GCT GCA GGT CGA CTC TAG ATC ATC CTC CGT AGA CCA CGG T
P17	gib_tri_3xFLAG_f	GGA CAC CGT GGT CTA CGG AGG ATC TAG AGA CTA CAA AGA CCA TGA C
P18	gib_pRM4_Sav1866_f	ATA AGC TAG CCA GGG GAG GAC CCA TAT GAT TAA ACG ATA TTT GCA ATT TGT TAA GCC
P19	gib_pRM4_Sav1866_r	CTC GGG CTG CAG GTC GAC TCT AGA TTA TAA GTT TTG AAT GCT ATA TAA ATG CTC GTA AG
P20	gib_Sav1866_FLAG_f	AGG TGC TTA CGA GCA TTT ATA TAG CAT TCA AAA CTT ATC TAG AGA CTA CAA AGA CCA TGA C
P21	gib_FLAG_Sav1866_r	CAC CGT CAT GGT CTT TGT AGT CTC TAG ATA AGT TTT GAA TGC TAT ATA AAT GCT CGT AAG
P22	gib_pRM4_Tva_f	ATA AGC TAG CCA GGG GAG GAC CCA TAT GGA AGT GGT GTT GCG CTT C
P23	gib_pRM4_Tva_r	CTC GGG CTG CAG GTC GAC TCT AGA TCA TCC TCC GAA GCC AAT GG
P24	gib_FLAG_Tva_r	CAC CGT CAT GGT CTT TGT AGT CTC TAG ATC CTC CGA AGC CAA TGG GTT G
P25	gib_Tva_FLAG_f	GAT CCA ACC CAT TGG CTT CGG AGG ATC TAG AGA CTA CAA AGA CCA TGA C
P26	gib2_pRM4_Tba_f	ATA AGC TAG CCA GGG GAG GAC CCA TAT GGA CAT GGT GTT GCG TTT CGA G
P27	gib_TbaE545Q_r	GGG CGG TGG CTT GGT CGA GGA CGA CGA TC
P28	gib_Tba545Q_f	GAT CGT CGT CCT CGA CCA AGC CAC CGC CC
P29	gib_pRM4_3XFLAG_r	CTC GGG CTG CAG GTC GAC TCT AGA TCA TTT GTC ATC GTC ATC CTT GTA ATC
P30	gib_TbaR310A_r	CGG CCC GAA CAG CGC CTG GAG CAG GGT G
P31	gib_TbaR310A_f	CAC CCT GCT CCA GGC GCT GTT CGG GCC G
P32	gib_TbaQ309G_r	CGA ACA GCC GCC CGA GCA GGG TGG CGA TG
P33	gib_TbaQ309G_f	CAT CGC CAC CCT GCT CGG GCG GCT GTT CG
P34	gib_TbaT316A_r	CCC GGA CAG CTG GGC GAT CGG CCC GAA CA
P35	gib_TbaT316A_f	TGT TCG GGC CGA TCG CCC AGC TGT CCG GG
P36	gib_GSTurboID_Tba_r	GAG CGA TCA GCT TCA GAG GCA CAG TAT TGT CTT TGC TCC CTC CTC CGT AGC CCA TGT GTT GG
P37	gib_Tba_GSTurboID_f	CGG TGA TCC AAC ACA TGG GCT ACG GAG GAG GGA GCA AAG ACA ATA CTG TGC CTC TGA AGC
P38	gib_pRM4_His6_r	CAA GCT CGG GCT GCA GGT CGA CTC TAG ATC AGT GGT GGT GGT GGT GGT GCT C
P39	gib_pRM4_ABC30_f	ATA AGC TAG CCA GGG GAG GAC CCA TAT GAC CAT TGG AGA CGA CCC GAG C
P40	gib_GSTurboID_ABC30_r	GAG CGA TCA GCT TCA GAG GCA CAG TAT TGT CTT TGC TCC CGC CCA AAG CAC GGT TCC CGC
P41	gib_pRM4_TurboID_f	CCA AGA GCG GGA ACC GTG CTT TGG GCG GGA GCA AAG ACA ATA CTG TGC CTC TGA AGC

Table S5 | Strains used in this study

Strain	Relevant features	Reference
E. coli NEB5α	Cloning host for plasmid generation	New England Biolabs
E. coli NovaBlue	Cloning host for plasmid generation	Novagen®
E. coli JM110	*lacY dam dcm* [F’ *lacI*^{q} Z Δ *M15*] (Methylation deficient strain)	[97]

E. coli ET12567	F⁻ *dam*-13::Tn9 *dcm*-6 *hsdM hsdR* (Methylation deficient)	[98]
A. balhimycina DSM 44591	Balhimycin producing wild type	[99,100]
A. balhimycina Δ*tba*	*tba* deletion mutant	This study
A. balhimycina Δ*tba* [+*tba*]	Apr^R, *tba* deletion mutant, ΦC31attB(pRM4-*tba*)	This study
A. balhimycina Δ*tba* [+*tba-3xFLAG*]	Apr^R, *tba* deletion mutant, ΦC31attB(pRM4-*tba-3xFLAG*)	This study
A. balhimycina Δ*tba* [+*tri-3xFLAG*]	Apr^R, *tba* deletion mutant, ΦC31attB(pRM4-*tri-3xFLAG*)	This study
A. balhimycina Δ*tba* [+*tva-3xFLAG*]	Apr^R, *tba* deletion mutant, ΦC31attB(pRM4-*amoh-3xFLAG*)	This study
A. balhimycina Δ*tba* [+*sav1866-3xFLAG*]	Apr^R, *tba* deletion mutant, ΦC31attB(pRM4-*sav1866-3xFLAG*)	This study
A. balhimycina Δ*tba* [+*tbaE545Q-3xFLAG*]	Apr^R, *tba* deletion mutant, ΦC31attB(pRM4-*tbaE545Q-3xFLAG*)	This study
A. balhimycina Δ*tba* [+*tbaR310A-3xFLAG*]	Apr^R, *tba* deletion mutant, ΦC31attB(pRM4-*tbaR310A-3xFLAG*)	This study
A. balhimycina Δ*tba* [+*tbaQ309G-3xFLAG*]	Apr^R, *tba* deletion mutant, ΦC31attB(pRM4-*tbaQ309G-3xFLAG*)	This study
A. balhimycina Δ*tba* [+*tbaT316A-3xFLAG*]	Apr^R, *tba* deletion mutant, ΦC31attB(pRM4-*tbaT316A-3xFLAG*)	This study
A. balhimycina Δ*bhaA*	*bhaA* deletion mutant	[28]
A. balhimycina Δ*bgtfB*	*bgtfB* deletion mutant	This study
A. balhimycina Δ*bgtfB*Δ*bhaA*	*bhaA and bgtfB* double-deletion mutant	This study
A. balhimycina Δ*tba* [+*tba-TID*]	Apr^R, *tba* deletion mutant, ΦC31attB(pRM4-tba-*TID*)	This study
A. balhimycina [+*TID*]	ΦC31attB(pRM4-*TID*)	This study
B. subtilis DSM10	Indicator strain for bioactivity assay	[101]

*Apr^R (Apramycin resistance)

Table S6 | Plasmids used in this study

Plasmid	Annotation	Resistance	Reference
pRM4	pSET152*ermEp** derived Φ31 integration vector with artificial ribosomal binding site	Apr^R	[10]
pSP1	pT7/T3-α19 derived gene disruption vector, with *ermE* gene in *Sap*I site	Ery^R/ Amp^R	[57]
pSP1Δ*tba*	pSP1 derived deletion vector for *tba* gene	Ery^R/ Amp^R	This study
pSP1Δ*bgtfB*	pSP1 derived deletion vector for *bgtfB* gene	Ery^R/ Amp^R	This study
pRM4-tba	pRM4 derived integrative *tba* expression vector	Apr^R	This study
pRM4-tba-3xFLAG	pRM4 derived integrative *tba-3xFLAG* expression vector	Apr^R	This study
pRM4-tri-3xFLAG	pRM4 derived integrative *tri-3xFLAG* expression vector	Apr^R	This study
pRM4-tva-3xFLAG	pRM4 derived integrative *tva-3xFLAG* expression vector	Apr^R	This study
pRM4-sav1866-3xFLAG	pRM4 derived integrative *sav1866-3xFLAG* (multidrug exporter of *S. aureus*) expression vector	Apr^R	This study
pRM4-tbaE545Q-3xFLAG	pRM4 derived integrative *tbaE545Q-3xFLAG* expression vector	Apr^R	This study
pRM4-tbaR310A-3xFLAG	pRM4 derived integrative *tbaR310A-3xFLAG* expression vector	Apr^R	This study
pRM4-tbaQ309G-3xFLAG	pRM4 derived integrative *tbaQ309G-3xFLAG* expression vector	Apr^R	This study
pRM4-tbaT316A-3xFLAG	pRM4 derived integrative *tbaT316A-3xFLAG* expression vector	Apr^R	This study
pET21a-TurboID-His6 (Addgene #107177)	TurboID expression vector; under control of P_{T7}	Amp^R	[31]
pRM4-tba-TID	pRM4 derived integrative *tba-TID* expression vector	Apr^R	This study
pRM4-TID	pRM4 derived integrative *TID* expression vector	Apr^R	This study
pRM4-abc30-TID	pRM4 derived integrative *abc30-TID* expression vector	Apr^R	This study

*Apr^R (Apramycin resistance), *Ery^R (Erythromycin resistance),*Amp^R (Ampicillin resistance)

References supplementary information

1. Hessa, T. *et al.* Molecular code for transmembrane-helix recognition by the Sec61 translocon. *Nature* **450**, 1026–1030 (2007).
